# An NHC‐Mediated Metal‐Free Approach towards an NHC‐Coordinated Endocyclic Disilene

**DOI:** 10.1002/open.202100240

**Published:** 2022-02-09

**Authors:** Thomas Lainer, Deepak Dange, Michael Pillinger, Roland C. Fischer, Anne‐Marie Kelterer, Cameron Jones, Michael Haas

**Affiliations:** ^1^ Institute of Inorganic Chemistry Graz University of Technology Stremayrgasse 9/IV 8010 Graz Austria; ^2^ School of Chemistry Monash University PO Box 23 Australia; ^3^ Institute of Physical and Theoretical Chemistry Stremayrgasse 9/I 8010 Graz Austria

**Keywords:** carbenes, disilene, silanes, silicon frameworks, subvalent compounds

## Abstract

A convenient metal‐free approach towards an N‐heterocyclic carbene (NHC)‐coordinated disilene **2** is described. Compound **2**, featuring the disilene incorporated in cyclopolysilane framework, was obtained in good yield and characterized using NMR spectroscopy and X‐ray crystallography. Density functional theory (DFT) calculations of the reaction mechanism provide a rationale for the observed reactivity and give detailed information on the bonding situation of the base‐stabilized disilene. Compound **2** undergoes thermal or light‐ induced (λ=456 nm) NHC loss, and a dimerization process to give a corresponding dimer with a Si_10_ skeleton. In order to shed light on the dimerization mechanism, DFT calculations were performed. Moreover, the reactivity of **2** was examined with selected examples of transition metal carbonyl compounds.

## Graphical abstract

**A metal‐free approach** towards an endocyclic disilene **2** is reported. The mechanism towards **2** was studied using DFT calculations. Based on quantum chemical calculations, the bonding situation within the endocyclic disilene was determined. Moreover, compound **2** undergoes a thermal or light‐induced dimerization with NHC loss to the corresponding dimer. Selected transition metal carbonyl compounds of **2** have been be prepared.

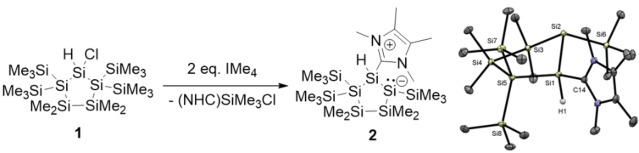

## Introduction

**Scheme 1:**
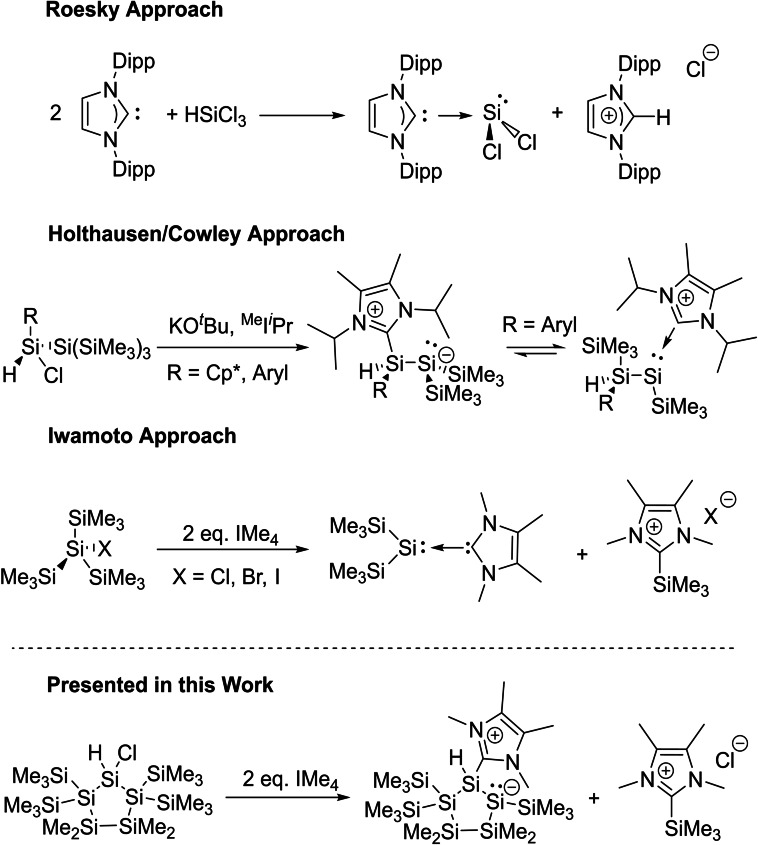
Selected examples of isolated base‐stabilized silylenes and the synthesis of a Lewis base‐stabilized endocyclic disilene.

The synthesis of base‐coordinated stable silylenes has attracted considerable attention in recent years. The groups of Roesky^[1]^ and Filippou^[2]^ pioneered the field with their simultaneous preparation of IPr⋅SiX_2_ (X=Cl, Br) using parallel strategies. In particular, the Roesky group used an innovative and mild synthetic strategy, which involves a reductive elimination of HCl from trichlorosilane in the presence of an NHC (N‐heterocyclic carbene; s. Scheme 1). This general strategy was used by many other groups and boosted the number of available silylenes significantly.^[3]^

Moreover, on the basis of this general strategy, a lot of emphasis was put into the synthesis and the reactivity of base stabilized silylenes.[^4^, ^5^] As a result, a variety of novel reactive species, which were previously elusive for silicon chemists, could now be intercepted as stable complexes.

Based on this, Cowley, Holthausen and coworkers investigated the equilibrium between disilenes (R_2_Si=SiR_2_) and their silylsilylenes isomers. The presence of NHCs during the reaction enabled the direct observation of the disilene/silylsilylene equilibrium by NMR spectroscopy. Moreover, all compounds involved in the equilibrium were structurally characterized (Scheme 1).^[6]^

Recently, the group of Iwamoto introduced a new approach towards NHC‐coordinated bis(trimethylsilyl)silylenes (Scheme 1).^[7]^ They reacted (tristrimethylsilyl)halosilanes with two equivalents of 1,3,4,5‐tetramethylimidazol‐2‐ylidene (IMe_4_) and observed the formation of an unstable base stabilized bis(silyl)‐silylene and the corresponding (NHC)SiMe_3_X salt. This indicates that the NHC reacts as a nucleophilic base to abstract trimethylhalosilane as well as like a Lewis base to stabilize the silylene. This is a remarkable finding, because all previous investigations showed that NHCs react under reductive elimination of HCl.

Similar to silylenes, a vast number of structural motifs incorporating a Si=Si unit have been published.^[8]^ Nevertheless, only a handful of anionic species of this type, so‐called disilenides, are available to date. In particular, the groups of Scheschkewitz,^[9]^ Sekiguchi^[10]^ and Iwamoto^[11]^ contributed to the synthesis of disilenyl anions and investigated their reactivity. Recently, several NHC‐coordinated cyclic silicon compounds were reported by the groups of Scheschkewitz, Jutzi and Lips.^[12]^ However, the synthesis of these NHC‐stabilized trisilacyclopropylidenes was either achieved by subsequent reaction of the disilenes with equimolar amounts of the carbene, or through metal‐induced (M=Li, Na, K, Mg, etc.) co‐reduction reactions of NHC‐adducts of silicon(IV) precursors. A completely metal‐free approach towards an NHC‐coordinated endocyclic functionalized disilene, which is incorporated in a cyclopolysilane framework, was not accomplished so far.

## Results and Discussion

**Scheme 2:**
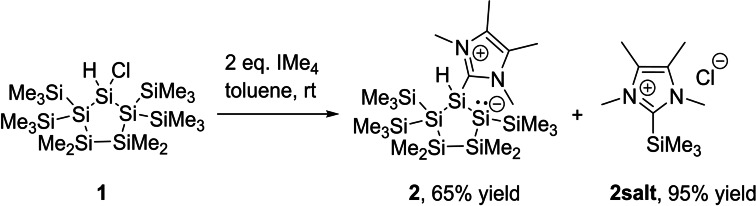
Synthesis of compounds **2** and **2 salt**.

Based on this progress in the field of silicon chemistry, we considered 1‐chloro‐3,3,4,4‐tetramethyl‐2,2,5,5‐tetrakis‐(tri‐methylsilyl)cyclopentasilane (**1**) as an ideal starting point for our chemical manipulations,^[13]^ as this new chemistry was never tested on cyclic polysilanes so far. Therefore, we reacted **1** with 2 equivalents of the Dipp‐carbene [:C{(DippNC)_2_}, Dipp=2,6‐diisopropylphenyl]. At room temperature, we detected no reaction, but at 80 °C in toluene an unselective reaction was observed leading to the complete degradation of the polysilane skeleton. Additionally, we tested the smaller NHC IMe_4_. We found a very fast and selective reaction to the base‐stabilized disilene **2** at room temperature (Scheme 2). **2** was isolated as a bright yellow powder in 65 % yield and purified by washing with *n*‐pentane. As a second product, we identified the corresponding (NHC)SiMe_3_Cl salt, which was recovered by filtration in 95 % yield. This indicates that the reactivity of IMe_4_ can be used as a general approach for the silyl abstraction of halopolysilanes, and that this methodology can be a useful alternative synthetic strategy for the formation of low valent group 14 derivatives.

**Figure 1:**
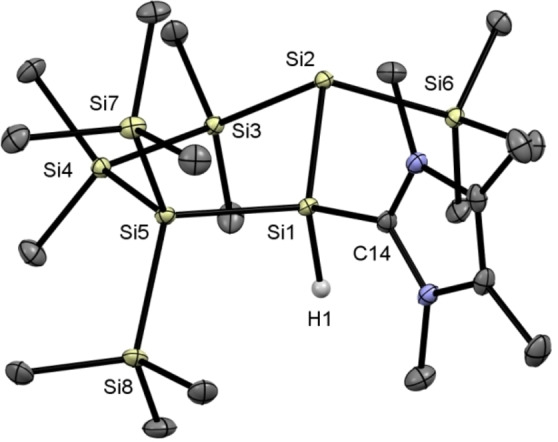
ORTEP representation of **2**. Thermal ellipsoids are drawn at the 30 % probability level. Hydrogen atoms (except the hydride) are omitted for clarity. Selected bond lengths (Å) and bond angles (°) with estimated standard deviations: Si(1)−Si(2) 2.3276(6), Si(1)−Si(3) 2.3542(6), Si(3)−Si(4) 2.3647(6), Si(4)−Si(5) 2.3528(6), Si(5)−Si(2) 2.3465(6), Si(1)−C(14) 1.9284(15), Si(1)−Si(2)−Si(5) 88.53(2), Si(1)−Si(2)−Si(6) 95.46(2), Si(5)−Si(2)−Si(6) 107.80(2), Si(2)−Si(1)−Si(3) 108.81(2), C(14)−Si(1)−Si(2) 115.83(5), C(14)−Si(1)−Si(3) 110.35(5).

Single crystals of **2** suitable for X‐ray structural analysis could be grown by cooling a concentrated solution of **2** in *n*‐pentane to −30 °C. The molecular structure is depicted in Figure 1. Similar to the base‐coordinated disilenes reported by Holthausen and Cowley et al.^[6]^, a long Si1−Si2 bond distance of 2.3276(6) Å indicates the presence of a single bond. Additionally, Si2 is significantly pyramidalized (sum of angles=292°), which indicates the presence of a lone pair of electrons and a formal negative charge at this silicon atom. Moreover, the cyclopentasilane ring adopts an envelope conformation commonly found for 5‐membered cyclosilanes.^[14]^

**Figure 2:**
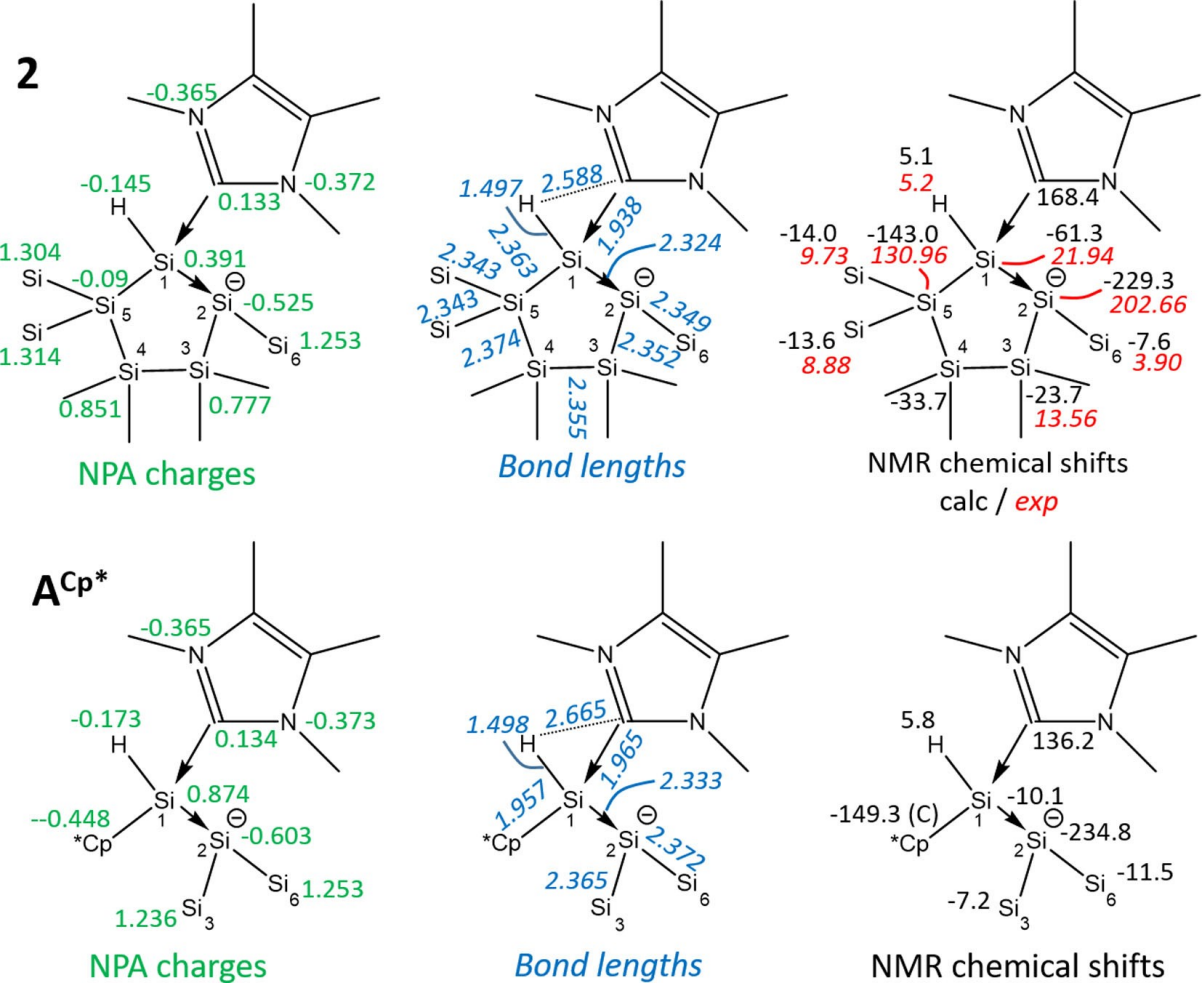
Comparison of NPA charges (green), bond lengths (in Å, blue) and computed (black) and experimental (in red, italic) NMR shifts (in ppm) of **2** (top) with A^Cp*^ (bottom). The computations were performed in the gas phase with the PBEh‐3c method. The atom numbering of A^Cp*^ is adapted to the atom numbering of **2**.

**Scheme 3:**
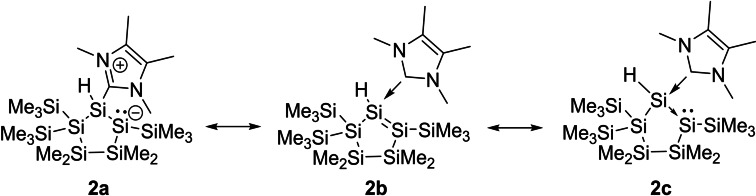
Lewis structure representations of disilene **2**.

Analytical data are consistent with the proposed structure. The ^29^Si NMR spectrum exhibits three signals for the magnetically inequivalent three SiMe_3_ groups between δ −3.93 and −9.72 ppm, two signals for the two endocyclic SiMe_2_ groups at δ −13.59 and −21.98 ppm, one signal for the hydride substituted silicon atom at δ −52.84 ppm, one signal for the quaternary silicon atom at δ −130.94 ppm, and one signal at δ −202.66 ppm for the three‐coordinated silicon atom. This high field resonance for Si2 in the ^29^Si NMR spectrum also indicates a negative charge, as this shift is typical for silyl anions.^[15]^ Moreover, we calculated the NMR shifts for compound **2** and found a good agreement between experimental and calculated values (Figure 2). Scheme 3 shows the three major Lewis resonance structure representations of compound **2**, zwitterionic **2 a**, disilene adduct **2 b**, or with dative C−Si and Si−Si bonds (**2 c**), which is reminiscent of published amine‐coordinated disilene Me_2_EtN→SiCl_2_→Si(Si(Cl_3_)_2_.^[16]^ To evaluate the bonding situation in **2**, we performed density functional theory (DFT) calculations.

The topological analysis of the electron density applying Bader's atom in molecules method^[17]^ as well as the Natural Bond Orbital (NBO) and the Natural Population Analysis (NPA) were interpreted for **2** and compared with the isolable disilene by Holthausen, Cowley and coworkers A^Cp*^.^[6]^ For both compounds, similar bonding characteristics as well as nearly identical chemical shifts were found (see Figure 2). The single bond between Si1 and Si2 confirms the observed crystallographic data. The calculated NPA charge distributions on the carbene (negative charges at both NHC nitrogen atoms) and at the disilene (positive charge at Si1 and negative charges at Si2) match with resonance structure **2 c**. The topological analysis of the electron density confirms this fact (see detailed discussion in the Supporting Information). The Laplacian of **2** as well as of A^Cp*^ (Table S4) indicates a high charge concentration along the C_NHC_−Si1 and Si1−Si2 bonds. This is visualized in the 2D plot of the Laplacian (Figure S10) which shows positive values in the internuclear region and spreading around both nuclei Si1 and Si2. The bond critical points (bcps) are shifted toward Si2 in the Si1−Si2 bond and toward Si1 in the C_NHC_−Si1 bond (see Figure S11 and data in Table S10), indicating a dative bond. The charge distribution together with the bond lengths supported by the position of the bond critical points and the Laplacian show a dative bonding network C_NHC_→Si1→Si2.

**Figure 3:**
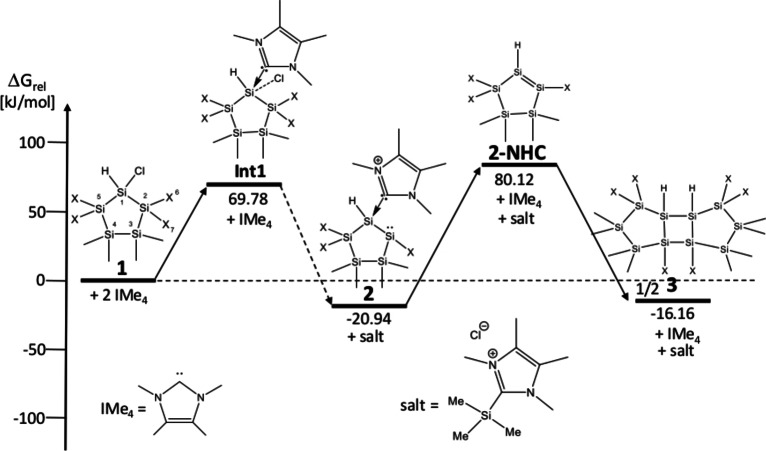
Thermodynamics of the reactions under investigation. X=SiMe_3_. The experimentally confirmed adduct **2** was formed by approaching IMe_4_ from the back side near H−Si1−Cl (intermediate 1), Compound **3** was formed after detachment of the NHC and subsequent dimerization.

As the chlorine as well as one silyl group are abstracted to form **2**, we performed DFT calculations to determine the reaction energies (see Figure 3). However, based on the high flexibility of the five‐membered ring and the sterically bulky trimethylsilyl‐groups, it was not possible to find the transition states for this new reaction. Consequently, we evaluated the approach of the carbene (IMe_4_) to the starting material from different sides (in the ring plane, from below or above) towards Si1 or towards Si2, forming three stable intermediates (**Int1**, **Int2**, **Int3**). The most important intermediate **Int1** (ΔG_rel_=69.78 kJ mol^−1^) forms a bond between the carbene and Si1 (r(Si1−C_carbene_)=1.933 Å), and at the same time, a chlorine atom is released from the educt and moves below the five‐membered ring (r(Si1−Cl=2.789 Å)). The other two intermediates form only loose interactions of the carbene with the starting material (**Int2**: r(H1−C_carbene_=2.923 Å and **Int3**, r(Si6−C_carbene_=4.195 Å), but no release of chlorine or the TMS groups occurs (for details, see the Supporting Information). The second IMe_4_ molecule releases one TMS group and chlorine from **Int1**, and the stable adduct **2** as well as the respective salt are formed. The Gibbs energy for the formation of **2** was computed to −20.94 kJ mol^−1^ relative to the starting material. The possible silyl abstraction from **Int2** shows a higher reaction Gibbs energy than the chlorine abstraction (ca. 400 kJ mol^−1^ compared to 215 kJ mol^−1^) when only the first carbene is included in the calculations.

**Scheme 4:**
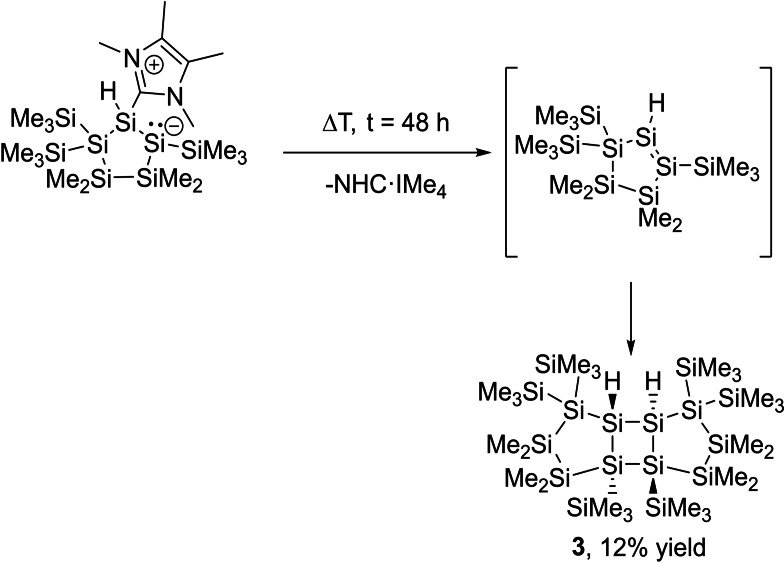
Proposed mechanism for the dimerization of **2**.

**Figure 4:**
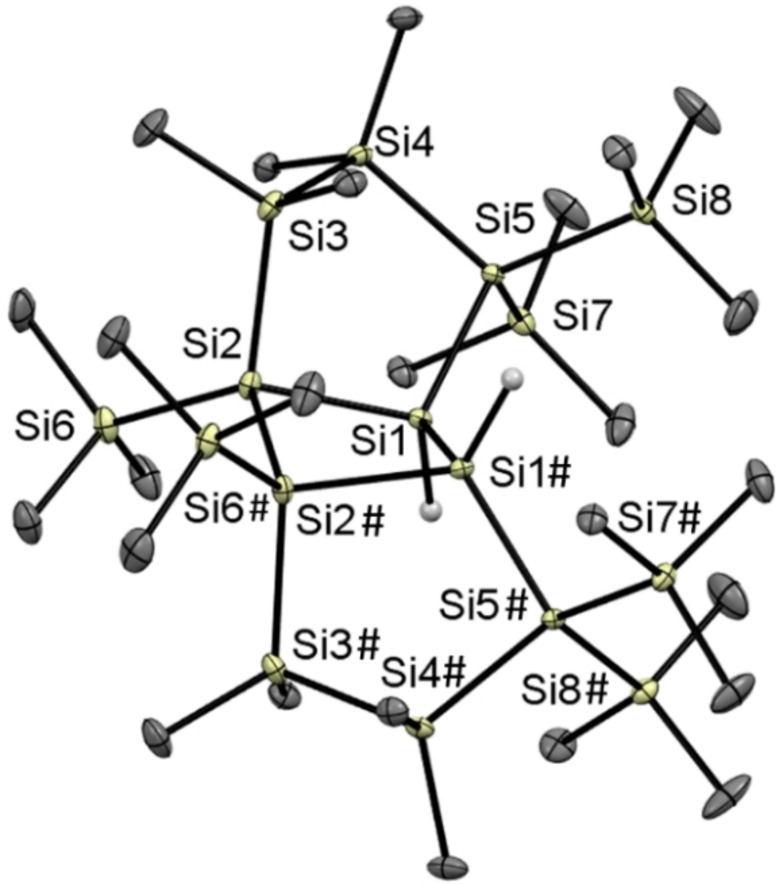
ORTEP representation of **3**. Thermal ellipsoids are drawn at the 30 % probability level. Hydrogen atoms (except the hydrides) are omitted for clarity. Selected bond lengths (Å) and bond angles (°) with estimated standard deviations: Si(1)−Si(1#) 2.3665(6), Si(1)−Si(5) 2.3751(4), Si(1)−Si(2) 2.3516(4), Si(2)−Si(3) 2.3512(4), Si(3)−Si(4) 2.3540(5), Si(5)−Si(5#) 2.3700(6).

This indicates that the second carbene plays a crucial role, which was also found by Iwamoto and coworkers. Alternative calculations of silyl abstraction at Si2 (with chlorine still attached at Si1 and the carbene attached at Si2) lead to a ring opening, which was not observed experimentally. Therefore, we conclude that this reaction proceeds through a carbene‐mediated chlorine abstraction and subsequent silyl abstraction. As outlined by Holthausen and Cowley, these NHC‐coordinated disilenes have the tendency to rearrange to the corresponding silylsilylenes. But this could not be confirmed by DFT calculations, which finds a high barrier of 68.9 kJ mol^−1^ (calculated without the attached carbene) for the rearrangement of product **2** to the respective silylene, rendering this rearrangement impossible in the framework of the fixed five‐membered ring. This was also verified by experiments. For this, we dissolved **2** in THF‐d_8_ and stirred this solution at room temperature for several days without detecting any reaction. We then raised the temperature to 60 °C and stirred this solution for an additional two days. After several hours, a colorless crystalline precipitate started forming. In addition, the reaction solution formed a significant amount of uncharacterisable polymer. The colorless precipitate was characterized by single crystal X‐ray diffraction (XRD) to be the dimerization product of **2**, after loss of coordinated IMe_4_ (see Scheme 4). The molecular structure and selected bond lengths are depicted in Figure 4. Compound **3** crystallized in the monoclinic space group *C2/c* with unexceptional bond lengths and angles. The unit cell contains four molecules (see Supporting Information). The low solubility of the dimer **3** prevented a complete characterization. However, in order to explain the formation of the head‐to‐head dimer **3**, DFT calculations were performed. Compound **2**, without the carbene, has a positive Gibbs energy (+80.6 kJ mol^−1^), which makes it thermodynamically unstable, whereas the head‐to‐head dimer is stabilized by −16 kJ mol^−1^. The second, higher relative energy of the head‐to‐tail dimer (+6.06 kJ mol^−1^) causes a relative distribution of the dominant head‐to‐head dimer of about 92 %. Therefore, this dimerization product was found in the reaction mixture (the calculated geometries can be found in the Supporting Information).

**Figure 5:**
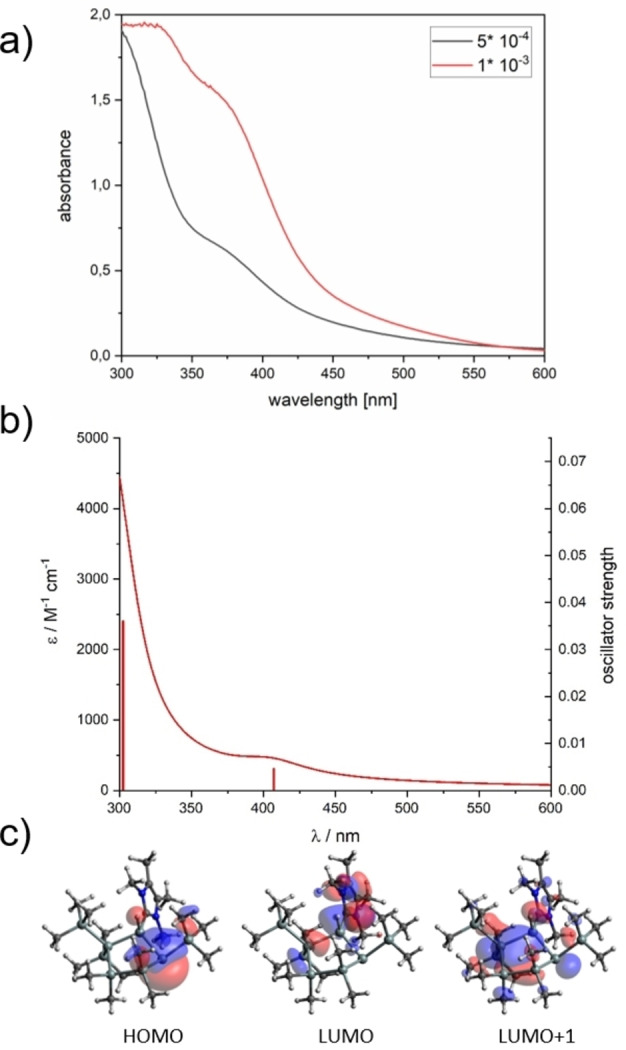
a) Experimental UV/Vis spectra of **2** in *n*‐hexane at two different concentrations and b) calculated absorption spectrum for compound **2**. The vertical transitions are marked as vertical lines with their respective oscillator strengths (right axis). The orbitals involved in the first transitions are presented in c) with a contour value of 0.04 a.u.

*n*‐Hexane was used as a solvent to determine the charge transfer behavior for the longest wavelength absorption bands of compound **2**. Figure 5 depicts the measured and calculated UV/Vis spectra together with their calculated frontier Kohn–Sham orbitals for the HOMO, LUMO and LUMO+1 orbitals. Compound **2** exhibits two absorption bands with *λ*_max_=300 nm (band I) and 380 nm (band II). Qualitative agreement between calculated and experimental absorption maxima could be achieved for both bands. In band II, consisting of the first excitation, the S1 transition is assigned to the HOMO‐LUMO excitation. The HOMO mainly corresponds to the p_z_ orbital of the silicon atom with little variation in shape and energy. Upon excitation, electron density is displaced into the π* orbital of the NHC moiety (LUMO). The second band S2 consists of excitations from HOMO into LUMO+1, representing the excitation into the σ* orbital of the silicon ring (see the detailed spectral data and the orbital picture in the Supporting Information).

Given the well‐known ability of some disilenes to activate normally inert substrates,^[18]^ we investigated reactions of C_6_D_6_ solutions of **2** with a range of gaseous substrates, and monitored the course of those reactions using NMR spectroscopy. When solutions of **2** were placed under atmospheres of H_2_, CO or ethylene, no reaction was observed under standard conditions. However, when those solutions were irradiated with blue light (λ=456 nm) overnight, the major, and only identifiable products, as determined by NMR spectroscopy, were **3** and free IMe_4_. In light of the relatively clean photochemical conversion of **2** to **3**, the photolysis experiment was repeated on a larger scale using a dinitrogen atmosphere. This gave **3** in a higher isolated yield (viz. 23 %) than for the aforementioned thermolysis experiment. Reaction of C_6_D_6_ solutions of **2** with either CO_2_ or N_2_O also yielded dimer **3**, with the by‐products being the known IMe_4_⋅CO_2_ adduct,^[19]^ and the IMe_4_⋅N_2_O adduct, which was identified by an X‐ray crystallographic study.

**Scheme 5:**
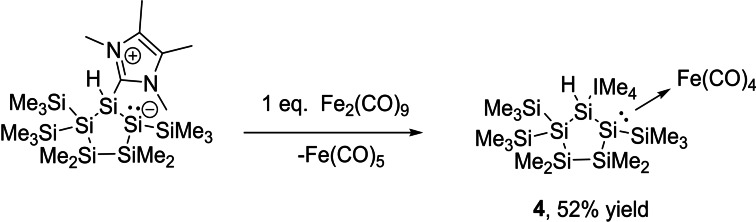
Synthesis of iron carbonyl complex **4**.

**Figure 6:**
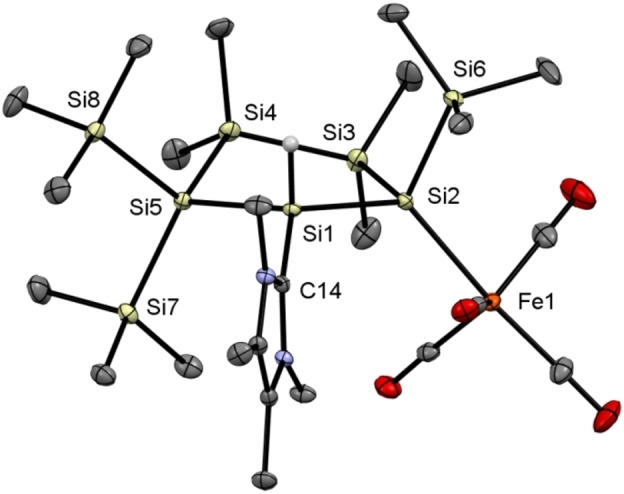
ORTEP representation of **4**. Thermal ellipsoids are drawn at the 30 % probability level. Hydrogen atoms (except the hydride) are omitted for clarity. Selected bond lengths (Å) and bond angles (°) with estimated standard deviations: Si(1)−Si(2) 2.3837(8), Si(1)−Si(5) 2.3558(8), Si(2)−Si(3) 2.3791(8), Si(3)−Si(4) 2.3565(9), Si(4)−Si(5) 2.3596(8), Si(1)−C(14) 1.916(2), Si(5)−Si(1)−Si(2) 114.52(3), Si(1)−Si(2)−Si(3) 99.87(3), C(14)−Si(1)−Si(2) 119.61(7), C(14)−Si(1)−Si(5) 110.78(6).

To test the donor acceptor properties of **2**, its transformation into silyl‐transition metal carbonyl complexes was performed and the products were subsequently characterized by standard analytical methods. For base‐coordinated silylenes, numerous transition metal complexes have already been reported.[^5^, ^20^, ^21^, ^22^] The entry into this chemistry is provided by the straightforward reaction of **2** with Fe_2_(CO)_9_, which resulted in the formation of **4** in 52 % yield (Scheme 5). The ^29^Si NMR spectroscopic resonance for the Si2 atom is detected at δ=−71.5 ppm and, upon coordination of the Fe(CO)_4_ unit, is shifted to lower field by 131.2 ppm relative to the resonance in uncoordinated **2**. The four carbonyl ligands show one singlet in the ^13^C NMR spectrum at a chemical shift of δ=217.7 ppm, which is caused by the Berry pseudorotation mechanism. All other resonances have been assigned and can be found in the Experimental Section. Single crystals of **4** suitable for X‐ray structure analysis were grown by cooling a concentrated solution of **4** in THF to −30 °C. The molecular structure is depicted in Figure 6. The Si2−Fe1 bond length (2.3781(7) Å) in **4** is in the upper region in comparison to other reported silylene‐Fe(CO)_4_ complexes (2.196–2.372 Å).[^5^, ^14^] However, similar Si−Fe bond lengths have been reported for silyl anion iron complexes as well as for trisilacyclopropylidene iron complexes (2.34–2.40 Å).[^22^, ^23^]

**Scheme 6:**
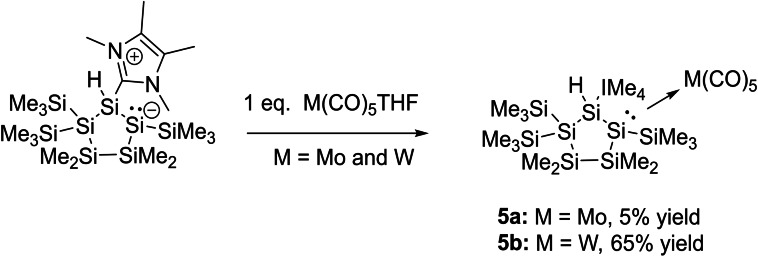
Synthesis of molybdenium and tungsten pentacarbonyl complexes **5 a**,**b**.

The reaction of **2** with equimolar amounts of a freshly prepared solution of M(CO)_5_⋅THF (M=Mo and W, synthesized by the photolysis of the corresponding hexacarbonyl metal complexes in THF) resulted in the formation of the pentacarbonyl tungsten complex **5 a** in good yields, and of the pentacarbonyl molybdenum complex **5 b** in very low yields (Scheme 6).

**Scheme 7:**
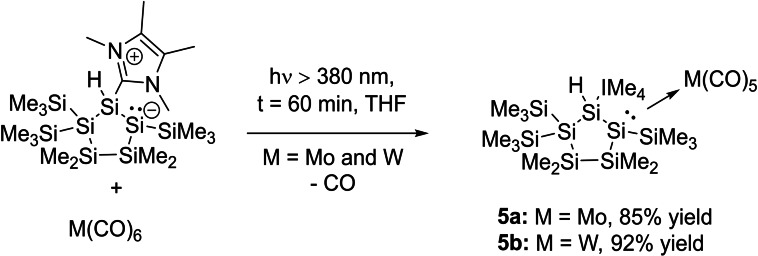
Photolysis of **2** in the presence of equimolar amounts of M(CO)_6_ (M=Mo and W).

On the basis of the low selectivity of the photolysis reactions giving pentacarbonyl molybdenum and tungsten THF complexes,^[22]^ we also investigated the simultaneous photolysis of **2** with M(CO)_6_ (M=Mo and W) in THF (Scheme 7). Interestingly, we found a very selective reactivity, and both compounds (**5 a**,**b**) were now isolable nearly quantitatively. This also demonstrates the good donor ability of **2**, as it selectively reacts with the in situ‐generated pentacarbonyl THF metal complexes and forms the adducts **5 a**,**b**. Intriguingly, compound **3** was not observed as a product of these photolysis reactions, despite the aforementioned generation of **3** upon irradiation of **2** in the absence of metal carbonyls.

**Figure 7:**
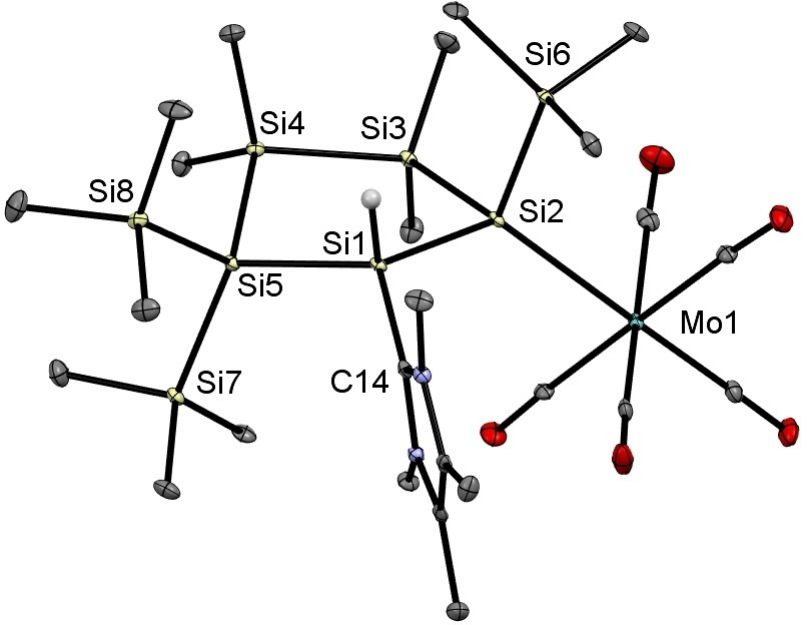
ORTEP representation of **5 a**. Thermal ellipsoids are drawn at the 30 % probability level. Hydrogen atoms are omitted for clarity. Selected bond lengths (Å) and bond angles (°) with estimated standard deviations: Mo(1)−Si(2) 2.6629(8), Si(1)−Si(2) 2.3659(10), Si(1)−Si(5) 2.3694(9), Si(3)−Si(4) 2.3542(10), Si(4)−Si(5) 2.3651(10), Si(1)−C(14) 1.923(3), Si(5)−Si(1)−Si(2) 115.75(4), Si(1)−Si(2)−Si(3) 98.63(3), C(14)−Si(1)−Si(2) 114.48(9), C(14)−Si(1)−Si(5)114.72(8).

**Figure 8:**
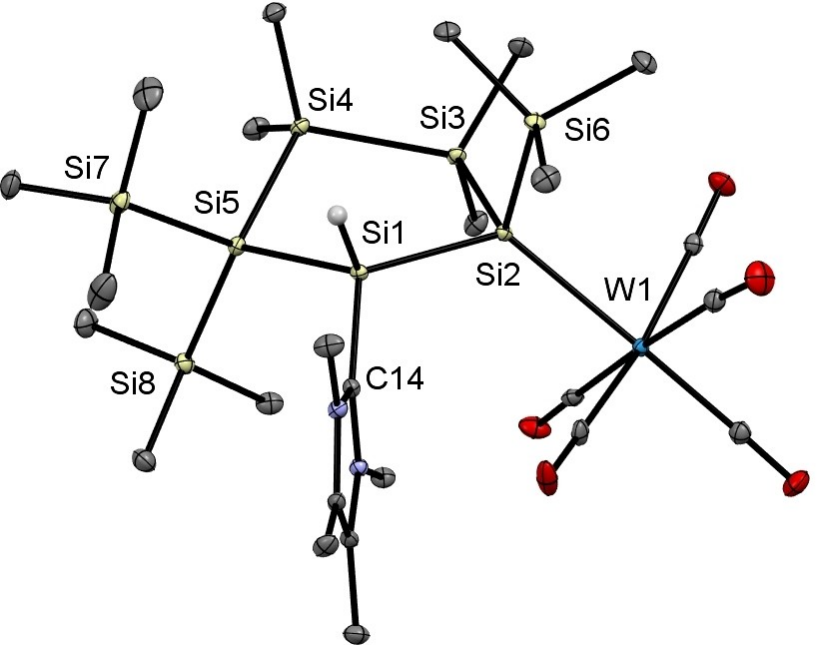
ORTEP representation of **5 b**. Thermal ellipsoids are drawn at the 30 % probability level. Hydrogen atoms are omitted for clarity. Selected bond lengths (Å) and bond angles (°) with estimated standard deviations: W(1)−Si(2) 2.6594(4), Si(1)−Si(2) 2.3722(6), Si(1)−Si(5) 2.3715(6), Si(3)−Si(4) 2.3582(6), Si(4)−Si(5) 2.3639(6), Si(1)−C(14) 1.9163(15), Si(5)−Si(1)−Si(2) 115.78(2), Si(1)−Si(2)−Si(3) 97.75(2), C(14)−Si(1)−Si(2) 115.41(5), C(14)−Si(1)−Si(5) 112.25(5).

Single crystals suitable for XRD measurements were obtained by cooling concentrated solutions of **5 a**,**b** in THF to −30 °C. Single crystal X‐ray analysis for both compounds revealed that the transition metals adopt a close‐to‐octahedral coordination sphere with five terminal carbonyl ligands (Figures 7 and 8). The Si2−Mo bond length of 2.6629(7) Å is at the upper end of the reported range for Si−Mo bond lengths (2.41–2.71 Å).[^21^, ^24^] In close analogy, the Si2−W bond of 2.6594(4) Å is also long in comparison to previously published examples (2.34–2.67 Å).[^21^, ^22^, ^25^] Moreover, it is in the range of Si−W single bond lengths (2.69 Å mean) measured in silanide complexes, for example [(SiMe_3_)_3_Si−W(CO)_5_]^−^.^[26]^

The ^13^C NMR spectra of **5 a**,**b** revealed two signals for the five carbonyl atoms, due to the axial and equatorial positions of the carbonyl groups. All other resonances have also been assigned and can be found in the Experimental Section.

## Conclusion

In conclusion, we have introduced a synthetic strategy yielding a novel coordinated endocyclic disilene **2**. This disilene was characterized by multinuclear NMR spectroscopy as well as by X‐ray crystallography. DFT calculations of the reaction mechanism provide a rationale for the observed reactivity and detailed information on the bonding situation within the base‐stabilized disilene. Furthermore, we investigated the stability of **2** and found that this compound can undergo a dimerization process, induced either thermally or photochemically, forming the corresponding dimer **3** in addition to a NHC loss. In order to shed light on the mechanism of the formation of **3**, DFT calculations were performed, which revealed the thermodynamic driving force of this dimerization. Finally, the straightforward reaction of **2** with selected examples of transition metal carbonyls provided new tetracarbonyl iron‐silyl complexes as well as new pentacarbonyl molybdenum‐ and tungsten‐silyl complexes, in good to excellent yields. This study also demonstrates the generality of the carbene‐induced trimethylhalosilane abstraction of halopolysilanes. Further studies to probe the scope of this chemistry are currently in progress.

## Experimental Section

### General Considerations

All experiments were performed under a dinitrogen atmosphere using standard Schlenk techniques. Solvents were dried using a column solvent purification system.^[27]^ Commercial reagents were used as purchased unless otherwise noted. ^1^H (299.95 MHz), ^13^C (75.43 MHz) and ^29^Si (59.59 MHz) NMR spectra were either recorded on a Varian INOVA MHz 300 or a Varian Mercury MHz 300 spectrometer in C_6_D_6_ or THF‐d_8_ solutions and referenced to TMS using the internal ^2^H lock signal of the solvent. 1‐chloro‐3,3,4,4‐tetramethyl‐2,2,5,5‐tetrakis (trimethylsilyl)cyclopenta‐silane^[13]^ and 1,3,4,5‐tetramethylimidazol‐2‐ylidene (IMe_4_)^[28]^ were synthesized according to published procedures. HRMS spectra were recorded on a Kratos Profile mass spectrometer. Infrared spectra were obtained on a Bruker Alpha‐P Diamond ATR Spectrometer from the solid sample. Melting points were determined using a Buechi 535 apparatus and are reported uncorrected. Elemental analyses were carried out on a Hanau Vario Elementar EL apparatus. Irradiations were carried out using a Kessil PR160L blue (λ=456 nm, 50 W) light LED lamp, with the reaction vessel placed approximately 2 cm from the light source, whilst being cooled by an external fan.

### Synthesis of 2

500 mg (0.943 mmol, 1.00 eq.) of **1** were dissolved in 10 mL of toluene. Subsequently, 48.4 mg (1.982 mmol, 2.10 eq.) of IMe_4_ dissolved in 20 mL of toluene were added and stirred at room temperature for an additional 30 min. During this time, a colorless precipitate was formed and the solution became orange. After this time, the precipitate was filtered off and washed with three times with toluene. The colorless precipitate was characterized as the NHC⋅SiMe_3_Cl salt **2 salt**. Drying under vacuum (0.02 mbar) afforded 209 mg (95 %) of **2 salt**. The solvent of the filtrate was removed under vacuum (0.02 mbar) and crystallization of the residue from an *n*‐pentane solution at −30 °C afforded 335 mg (65 %) of **2**.

**2**: mp: 132–135 °C. Anal. Calcd. for C_20_H_52_N_2_Si_8_: C, 44.05; H, 9.61 %. Found.: C, 44.12; H, 9.55 %. ^29^Si NMR (C_6_D_6_, TMS, ppm): −3.93, −8.88, −9.72 (*Si*Me_3_); −13.59, −21.98 (*Si*Me_2_); −52.84 (*Si*H); −130.94 (*Si*_q_); −202.66 (*Si*SiMe_3_). ^13^C NMR (C_6_D_6_, TMS, ppm): 157.87 (s, IMe_4_, *C*−Si); 126.59 (s, IMe_4_, *C*−CH_3_); 34.95 (s, IMe_4_, N−*C*H_3_);7.94 (s, IMe_4_, C*C*H_3_); 6.19, 3.26, 3.08 (s, (Si(*C*H_3_)_3_); 3.59, 1.43, −0.36, −1.62 (Si(*C*H_3_)_2_). ^1^H NMR (C_6_D_6_, TMS, ppm): 5.24 (s, 1H, ^1^ *J*_Si‐H_=162 Hz, Si‐*H*); 3.45 (s, 6H, N−C*H*_3_); 1.04 (s, 6H, C−C*H*_3_); 0.83, 0.82 (s, 6H each, Si(C*H*_3_)_2_); 0.65 (s, 15H, Si(C*H*_3_)_2_ and Si(C*H*_3_)_3_), 0.42, 0.03 (s, 9H each, Si(C*H*_3_)_3_). IR (neat): ν(C=O)=ν(Si−H)=2076 (m) cm^−1^. HRMS: calc. for [C_20_H_52_N_2_Si_8_]^+⋅^ (M^+^): 544.2285; found: 544.2294. UV/Vis: λ [nm] (ϵ [L mol^−1^ cm^−1^])=355 (1420), 414 (653).

**2 salt**: mp: 138–141 °C. Anal. Calcd. for C_10_H_21_ClN_2_Si: C, 51.59; H, 9.09 %. Found.: C, 51.69; H, 9.17 %. ^29^Si NMR (CDCl_3_, TMS, ppm): 21.42 (*Si*Me_3_). ^13^C NMR (CDCl_3_, TMS, ppm): 126.68 (s, IMe_4_, *C*‐Si); 95.32 (s, IMe_4_, *C*−CH_3_); 33.66 (s, IMe_4_, N−*C*H_3_);8.34 (s, IMe_4_, C*C*H_3_); −4.07 (s, (Si(*C*H_3_)_3_). ^1^H NMR (CDCl_3_, TMS, ppm): 3.77 (s, 6H, N‐C*H*_3_); 2.14 (s, 6H, C−C*H*_3_); 0.21 (s, 9H, Si(C*H*_3_)_3_).

### Dimerization of 2

#### Thermolysis

70 mg (0.092 mmol) of **2** were dissolved in 1 mL of THF‐d_8_. Subsequently, this solution was stirred at 60 °C for an additional 48 h. During this time, a colorless precipitate was formed. At this point, the complete conversion of the starting material to a significant amount of uncharacterizable polymer was monitored by NMR spectroscopy. The colorless precipitate was filtered of and washed three times with THF. Drying under vacuum (0.02 mbar) afforded 4.6 mg (12 %) of **3**. The low solubility of **3** prevented a complete characterization by ^13^C NMR spectroscopy.

#### Photolysis

Compound **2** (75 mg, 0.1247 mmol) was dissolved in C_6_D_6_ (1 mL) in a J. Young's NMR tube. The sample was irradiated with blue light (456 nm) and the progress of the reaction monitored by ^1^H NMR spectroscopy. After 5 days, the sample showed complete conversion of the silylene to a new product with 12.0 mg (23 %) of colorless crystals of **3** having deposited from the solution.

**3: mp**: 290–292 °C. Anal. Calcd. for C_26_H_80_Si_16_: C, 37.08; H, 9.57 %. Found.: C, 37.37; H, 9.64 %. ^29^Si NMR (CDCl_3_, TMS, ppm): −7.02, −7.53, −10.37 (*Si*Me_3_); −20.97, −21.97, −23.69 (*Si*Me_2_); −100.85 (*Si*SiMe_3_); −134.72 (*Si*q). ^1^H NMR (CDCl_3_, TMS, ppm): 3.84 (s, 2H, Si‐*H*); 0.48, 0.44, 0.40, 0.07 (s, 6H each, Si(C*H*_3_)_2_); 0.24, 0.21, 0.18 (s, 9H each, Si(C*H*_3_)_3_). IR (neat): ν(Si−H)=2065 (m) cm^−1^.

#### Synthesis of 4

300 mg (0.550 mmol, 1.00 eq.) of **2** were dissolved in 10 mL of THF. Subsequently, 200 mg (1.982 mmol, 1.00 eq.) of [Fe_2_(CO)_9_] dissolved in 10 mL of THF, were added and the mixture was stirred at room temperature for an additional 18 h. During this time, a dark black precipitate formed and the solution became red. The complete conversion of the starting material to **4** was monitored by NMR spectroscopy. After this time, the precipitate was filtered off and washed three times with THF. Drying under vacuum (0.02 mbar) afforded 345 mg (88 %) of **4** as an orange solid.

**4**: mp: 198–200 °C. Anal. Calcd. for C_24_H_52_FeN_2_O_4_Si_8_: C, 40.42; H, 7.35 %. Found.: C, 40.57; H, 7.62 %. ^29^Si NMR (THF‐d_8_, TMS, ppm): −4.75, −7.12, −7.78 (*Si*Me_3_); −23.16, −27.14 (*Si*Me_2_); −67.39 (*Si*H); −71.79 (*Si*SiMe_3_); −129.35 (*Si*q). ^13^C NMR (THF‐d_8_, TMS, ppm): 218.88 (s, Fe(*C*O)_4_; 153.16 (s, IMe_4_, *C*‐Si); 130.54 (s, IMe_4_, *C*−CH_3_); 36.91 (s, IMe_4_, N−*C*H_3_); 8.75 (s, IMe_4_, C*C*H_3_); 3.58, 3.15, 3.01 (s, (Si(*C*H_3_)_3_); −0.48, −0.83, −1.38, −1.65 (Si(*C*H_3_)_2_). ^1^H NMR (C_6_D_6_, TMS, ppm): 4.82 (s, 1H, ^1^ *J*_Si‐H_=166 Hz, Si‐*H*); 3.92 (s, 6H, N−C*H*_3_); 2.26 (s, 6H, C−C*H*_3_); 0.49, 0.43, 0.37, 0.35 (s, 3H each, Si(C*H*_3_)_2_); 0.25 (s, 18H, Si(C*H*_3_)_3_), 0.20, (s, 9H Si(C*H*_3_)_3_). ν(Si−H)=1993 (m) cm^−1^; ν(C=O)=1909, 1878, 1861 (s) cm^−1^.

### Synthesis of 5 a

#### Method A

300 mg (0.550 mmol, 1.00 eq) of **2** were dissolved in 10 mL of THF. This solution was subsequently added to a freshly prepared THF solution of Mo(CO)_5_⋅THF (prepared from 146 mg (0.550 mmol, 1.00 eq.) of Mo(CO)_6_ dissolved in 5 mL THF and irradiated for 3 h). This solution was then allowed to stir for an additional 1 h at room temperature. The complete conversion of the starting material to **5 a**, alongside with undefined side products, was monitored by NMR spectroscopy. The solvent of the reaction solution was removed under vacuum (0.02 mbar) and the solid residue was washed three times with toluene. Drying under vacuum (0.02 mbar) afforded 22 mg (5 %) of **5 a** as a pale yellow solid.

#### Method B

300 mg (0.550 mmol, 1.00 eq.) of **2** and 146 mg (0.550 mmol, 1.00 eq.) of Mo(CO)_6_ were dissolved in 10 mL of THF (some hexacarbonyl molybdenum complex was not completely dissolved and remained as solid residue on the bottom of the flask). This solution was subsequently irradiated for 1 h. The complete conversion of the starting material to **5 a** was monitored by NMR spectroscopy. The solvent of the photolysis reaction was removed under vacuum (0.02 mbar) and the solid residue was washed three times with toluene. Drying under vacuum (0.02 mbar) afforded 366 mg (85 %) of **5 a** as a pale yellow solid.

**5 a**: mp: 190 °C (dec.). Anal. Calcd. for C_25_H_52_MoN_2_O_5_Si_8_: C, 38.43; H, 6.71 %. Found.: C, 38.41; H, 6.55 %. ^29^Si NMR (THF‐d_8_, TMS, ppm): −4.02, −7.53, −8.39 (*Si*Me_3_); −15.79, −26.20 (*Si*Me_2_); −59.73 (*Si*H); −133.49 (*Si*SiMe_3_); −128.33 (*Si*q). ^13^C NMR (THF‐d_8_, TMS, ppm): 215.41, 214.58 (s, Mo(*C*O)_5_); 154.74 (s, IMe_4_, *C*−Si); 129.07 (s, IMe_4_, *C*−CH_3_); 36.68 (s, IMe_4_, N−*C*H_3_); 8.64 (s, IMe_4_, C*C*H_3_); 3.79, 3.58, 3.29 (s, (Si(*C*H_3_)_3_); 1.14, −0.52, −1.07, −1.28 (Si(*C*H_3_)_2_). ^1^H NMR (C_6_D_6_, TMS, ppm): 4.91 (s, 1H, ^1^ *J*_Si‐H_=167 Hz, Si−*H*); 3.90 (s, 6H, N−C*H*_3_); 2.25 (s, 6H, C−C*H*_3_); 0.41, 0.41, 0.37, 0.36 (s, 3H each, Si(C*H*_3_)_2_); 0.26, 0.24, 0.14 (s, 9H each, Si(C*H*_3_)_3_). IR (neat): ν(Si−H)=2034 (m) cm^−1^; ν(C=O)=1890, 1865 (s) cm^−1^.

### Synthesis of 5 b

#### Method A

300 mg (0.550 mmol, 1.00 eq.) of **2** were dissolved in 10 mL of THF. This solution was subsequently added to a freshly prepared THF solution of W(CO)_5_ ⋅ THF (prepared from 194 mg (0.550 mmol, 1.00 eq.) of W(CO)_6_ dissolved in 5 mL THF and irradiated for 3 h). This solution was then allowed to stir for additional 25 min. at room temperature. The complete conversion of the starting material to **5 b** was monitored by NMR spectroscopy. The solvent of the reaction solution was removed under vacuum (0.02 mbar) and the solid residue was washed three times with toluene. Drying under vacuum (0.02 mbar) afforded 311 mg (65 %) of **5 b** as a pale yellow solid.

#### Method B

300 mg (0.550 mmol, 1.00 eq) of **2** and 194 mg (0.550 mmol, 1.00 eq.) of W(CO)_6_ were dissolved in 10 mL of THF (the hexacarbonyl tungsten complex was not completely dissolved, and partly remained as solid residue at the bottom of the flask). This solution was subsequently irradiated for 1 h. The complete conversion of the starting material to **5 b** was monitored by NMR spectroscopy. The solvent of the photolysis reaction was removed under vacuum (0.02 mbar) and the solid residue was washed three times with toluene. Drying under vacuum (0.02 mbar) afforded 521 mg (92 %) of **5 b** as a pale yellow solid.

**5 b**: mp: 220 °C (dec.). Anal. Calcd. for C_25_H_52_N_2_O_5_Si_8_W: C, 34.55; H, 6.03 %. Found.: C, 34.68; H, 6.15 %. ^29^Si NMR (THF‐d_8_, TMS, ppm): −3.65, −7.56, −8.31 (*Si*Me_3_); −16.27, −26.45 (*Si*Me_2_); −62.59 (*Si*H); −144.79 (*Si*SiMe_3_); −128.10 (*Si*q). ^13^C NMR (THF‐d_8_, TMS, ppm): 204.85, 204.36 (s, W(*C*O)_5_); 154.47 (s, IMe_4_, *C*−Si); 129.07 (s, IMe_4_, *C*−CH_3_); 36.89 (s, IMe_4_, N−*C*H_3_); 8.63 (s, IMe_4_, C*C*H_3_); 3.63, 3.57, 3.26 (s, (Si(*C*H_3_)_3_); 1.19, −0.51, −1.09, −1.31 (Si(*C*H_3_)_2_). ^1^H NMR (C_6_D_6_, TMS, ppm): 4.91 (s, 1H, ^1^ *J*_Si‐H_=166 Hz, Si−*H*); 3.90 (s, 6H, N−C*H*_3_); 2.25 (s, 6H, C−C*H*_3_); 0.42, 0.42 (s, 3H each, Si(C*H*_3_)_2_); 0.37, (s, 6H, Si(C*H*_3_)_2_); 0.26, 0.25 (s, 9H each, Si(C*H*_3_)_3_), 0.15, (s, 9H Si(C*H*_3_)_3_). IR (neat): ν(Si−H)=2032 (m) cm^−1^; ν(C=O)=1882, 1852 (s) cm^−1^.

### X‐ray Crystallography

All crystals suitable for single crystal X‐ray diffractometry were removed from a vial and immediately covered with a layer of silicone oil. A single crystal was selected, mounted on a glass rod on a copper pin, and placed in the cold N_2_ stream provided by an Oxford Cryosystems cryostream. XRD data collection was performed for all compounds on a Bruker APEX II diffractometer with use of Mo Kα radiation (λ=0.71073 Å) and a CCD area detector. Empirical absorption corrections were applied using SADABS.^[29]^ The structures were solved with use of the intrinsic phasing option in SHELXT and refined by the full‐matrix least‐squares procedures in SHELXL.^[30]^ The space group assignments and structural solutions were evaluated using PLATON.^[31]^ Non‐hydrogen atoms were refined anisotropically. Hydrogen atoms were located in calculated positions corresponding to standard bond lengths and angles. CIF files were edited, validated and formatted with the program Olex2.^[32]^

Deposition Numbers 2115666 (for **2**), 2115667 (for **3**), 2115668 (for **4**), 2115669 (for **5 a**), 2115670 (for **5 b**) contain the supplementary crystallographic data (excluding structure factors) for this paper. These data are provided free of charge by the joint Cambridge Crystallographic Data Centre and Fachinformationszentrum Karlsruhe Access Structures service.

### Computational Methods

Density Functional Theory (DFT) calculations were performed for all compounds on the reaction pathway from the starting material toward the product dimer including **2** and **3** as well as relevant intermediates. The PB3h‐3c method^[33]^ was used for optimizations and computation of the UV/Vis spectra. This method is a composite DFT method combining the PBE functional with the def2‐mSVP basis set with formal corrections for the basis set superposition error and dispersion correction with Becke–Johnson damping^[34]^ as well as the geometrical counterpoise correction^[35]^ for the basis set superposition error. All geometries were optimized in toluene, applying the Conductor‐like Polarizable Continuum Model (CPCM) for solvation, and a harmonic frequency calculation with the PBEh‐3c method was performed to confirm the geometries as minima at the potential surface. The thermochemical data were corrected by single point calculations in toluene with the M06‐2X functional using the def2‐TZVP basis set. All calculations were performed with the program ORCA4.2.1^[36]^ applying standard parameters.

The Natural Population Analysis (NPA) charges and the Natural Bonding Orbital (NBO) analysis was performed with the program Gaussian09.^[37].^The Laplacian was drawn with the program Multiwfn.^[38].^

NMR chemical shieldings were computed by the Gauge‐Independent Atomic Orbital (GIAO) method using the one parameter version of the PW91 DFT functional with the IGLO‐III basis set and the def2/JK auxiliary basis set. TMS was taken as reference to compute the NMR chemical shifts. The calculations were performed in the solvent benzene applying the CPCM as implemented in ORCA4.2.1.

## Conflict of interest

The authors declare no conflict of interest.

1

## Supporting information

As a service to our authors and readers, this journal provides supporting information supplied by the authors. Such materials are peer reviewed and may be re‐organized for online delivery, but are not copy‐edited or typeset. Technical support issues arising from supporting information (other than missing files) should be addressed to the authors.

Supporting Information

Supporting Information

## Data Availability

The data that support the findings of this study are available in the supplementary material of this article.
