# Supplementary material for: An NHC‐Mediated Metal‐Free Approach towards an NHC‐Coordinated Endocyclic Disilene
Source: ChemistryOpen. 2022 Feb 9;11(3):e202100240. doi: 10.1002/open.202100240 (PMC8889510; doi:10.1002/open.202100240)
Supplement: Supplementary file 2 — Supporting Information [file OPEN-11-e202100240-s002.pdf]

ChemistryOpen

Supporting Information

An NHC-Mediated Metal-Free Approach towards an NHC-Coordinated Endocyclic Disilene

Thomas Lainer, Deepak Dange, Michael Pillinger, Roland C. Fischer, Anne-Marie Kelterer,* Cameron Jones,* and Michael Haas*

Table of Content

Analytical Section 2

NMR-Spectroscopy 2

Figure S1: ^{1}H-, ^{13}C-, and ^{29}Si-NMR spectra of **2** (C_6D_6 solution, vs ext. TMS, ppm) 2

Figure S2: ^{1}H-, ^{13}C-, and ^{29}Si-NMR spectra of **2salt** ($CDCl_3$ solution, vs ext. TMS, ppm) 3

Figure S3: ^{1}H-, and ^{29}Si-NMR spectra of **3** ($CDCl_3$ solution, vs ext. TMS, ppm) 4

Figure S4: ^{1}H-, ^{13}C-, and ^{29}Si-NMR spectra of **4** (THF-d_8 solution, vs ext. TMS, ppm) 5

Figure S5: ^{1}H-, ^{29}Si- and ^{13}C-NMR spectra of **5a** (THF d_8 solution, vs ext. TMS, ppm) [* toluene and # *n*-pentane] 6

Figure S6: ^{1}H-, ^{29}Si- and ^{13}C-NMR spectra of **5b** (THF d_8 solution, vs ext. TMS, ppm) [* toluene and # *n*-pentane] 7

Computational data. 8

Intermediates of the reaction of 1 with NHCs. 8

Figure S7. Three stable intermediates were formed when IMe_4 approaches from different sides to the educt **1**. 8

Reaction Gibbs Energies. 9

Figure S8. The calculated reaction with Gibbs energies for the stable conformations from educt **1** towards the dimer **3**. 9

Rearrangement of **2** towards silylene**.** 10

Figure S9.. 10

Thermodynamics of the reaction from **1** to **2**. 11

Table S1. Reaction Gibbs Free energy of different abstraction reactions. 11

Table S2. Reaction Gibbs Free energy of different abstraction reactions.. 12

Bonding analysis of **2** compared to Cowley's compound $\mathbf{A^{Cp*}}$. 13

Table S3. Mayer population and NBO analysis of compound **2** and Cowleys $\mathbf{A^{Cp*}}$ 13

Table S4. Topological data of the electron density of **2**: 14

Figure S10. 2D plot of the Laplacian $\nabla^2\rho(r)$ for **2** (left) and $\mathbf{A^{Cp*}}$ (right) 14

Figure S11. Bond critical points (bcps) of **2** in orange; important bcps are intensified in black for better visibility.. 15

UV/Vis data of **2**. 15

Figure S12. Calculated vertical excitations of **2,** and simulated spectrum with a FWHH broadening of 3600 cm^{-1}. 15

Table S5. First three calculated vertical excitations of **2**. The orbitals refer to the MO picture in the main part of the manuscript. 16

NMR chemical shifts of 2. 16

Table S6. Comparison of calculated and experimental NMR shifts (in ppm). 16

Crystallographic Tables 17

References 17

Analytical Section

NMR-Spectroscopy

Figure S1: ^{1}H-, ^{13}C-, and ^{29}Si-NMR spectra of **2** (C_6D_6 solution, vs ext. TMS, ppm)

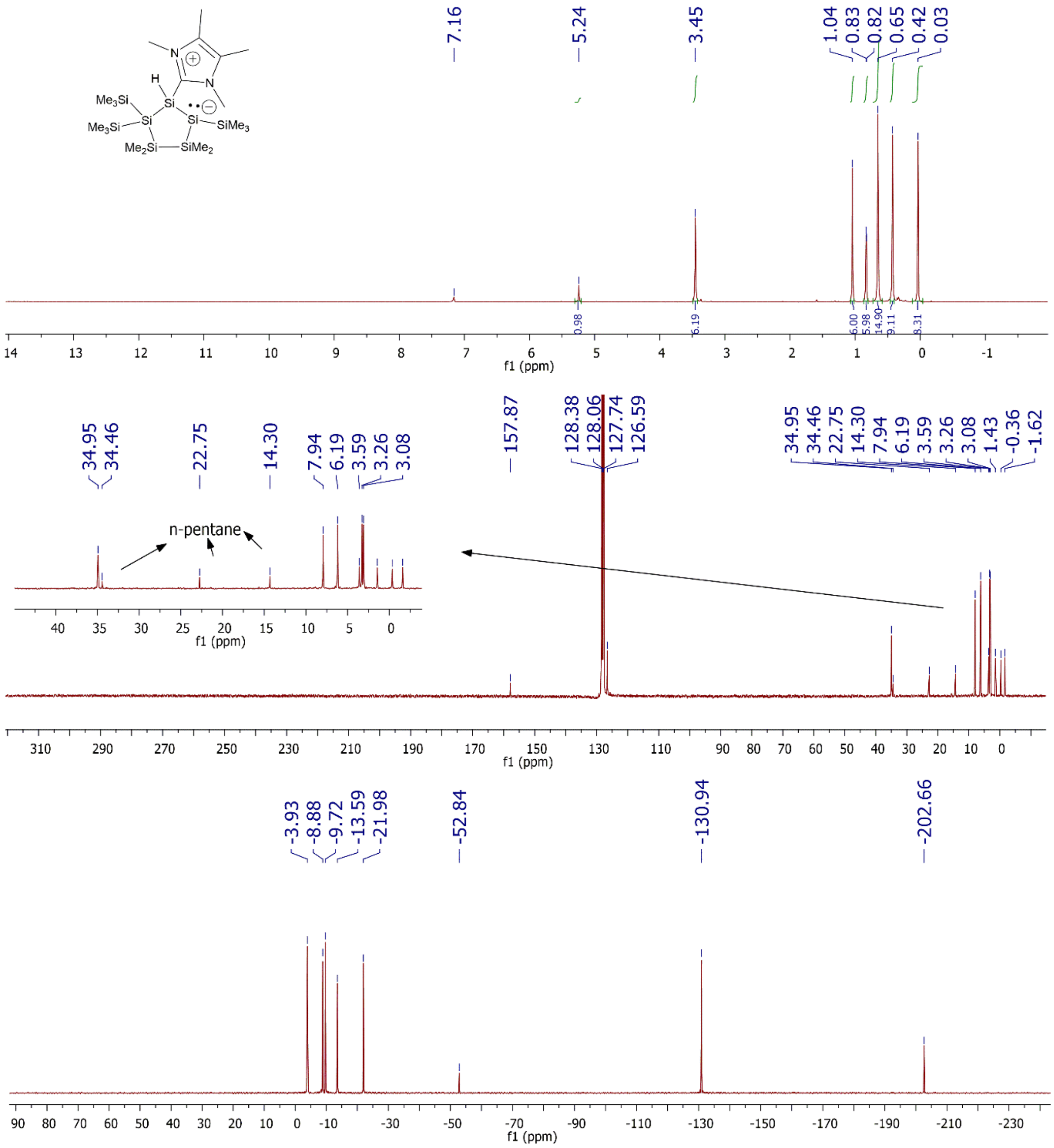

Figure S2: ^{1}H-, ^{13}C-, and ^{29}Si-NMR spectra of **2salt** ($CDCl_3$ solution, vs ext. TMS, ppm)

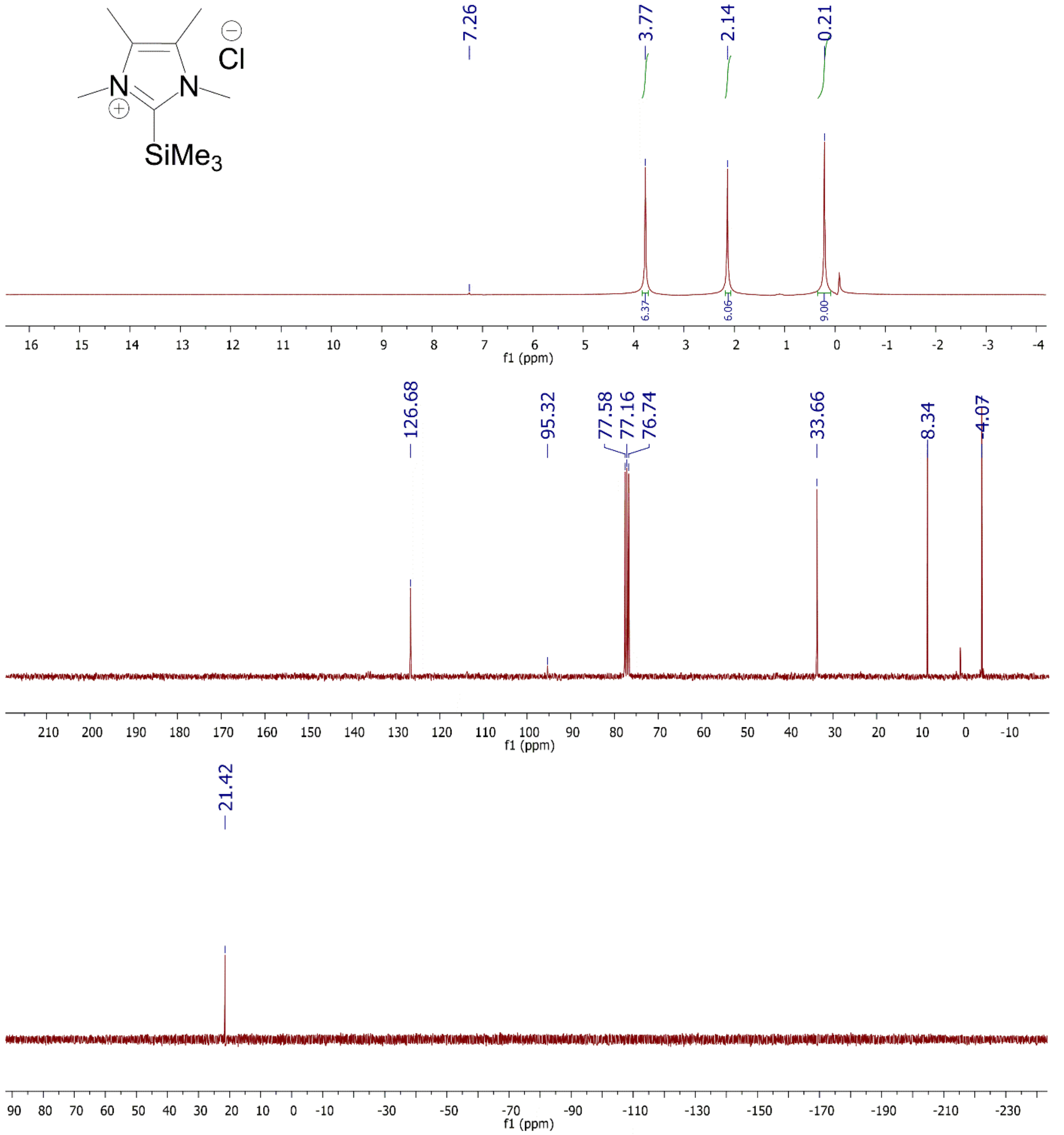

Figure S3: ^{1}H-, and ^{29}Si-NMR spectra of **3** ($CDCl_3$ solution, vs ext. TMS, ppm)

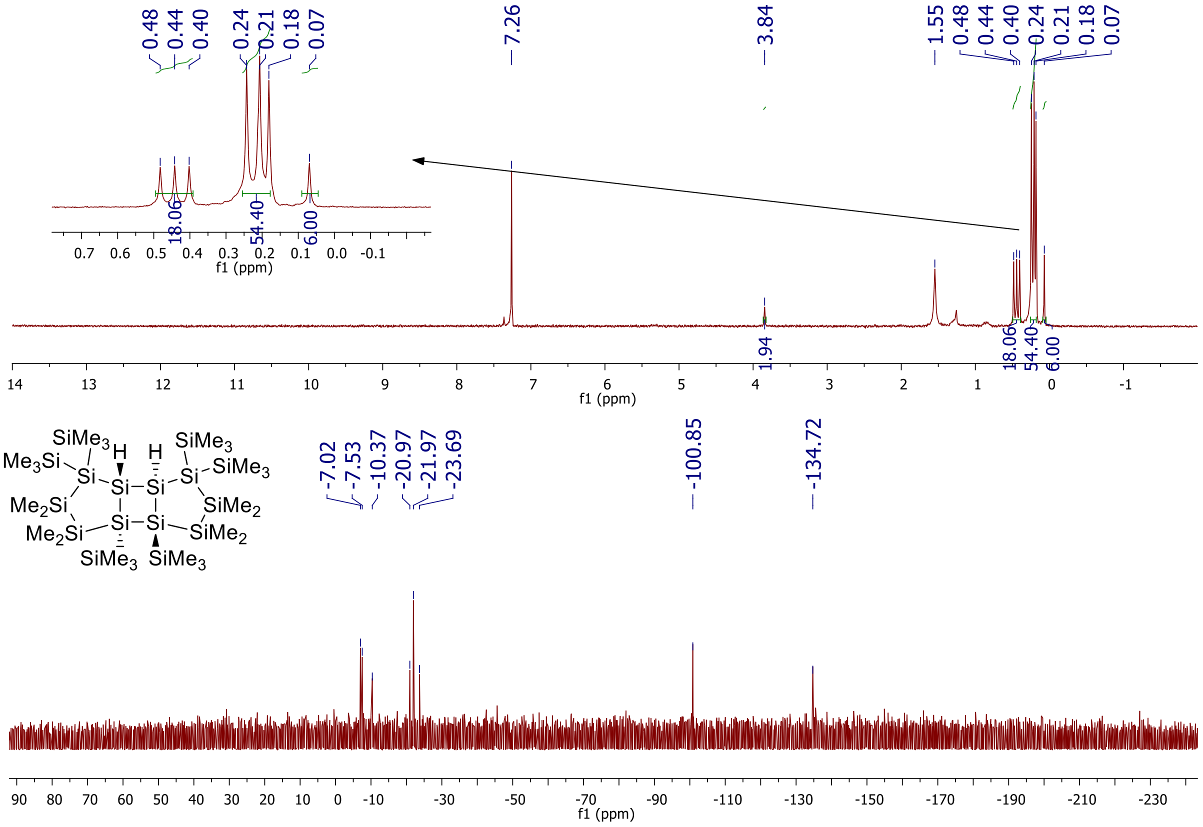

Figure S4: ^{1}H-, ^{13}C-, and ^{29}Si-NMR spectra of **4** (THF-d_8 solution, vs ext. TMS, ppm)

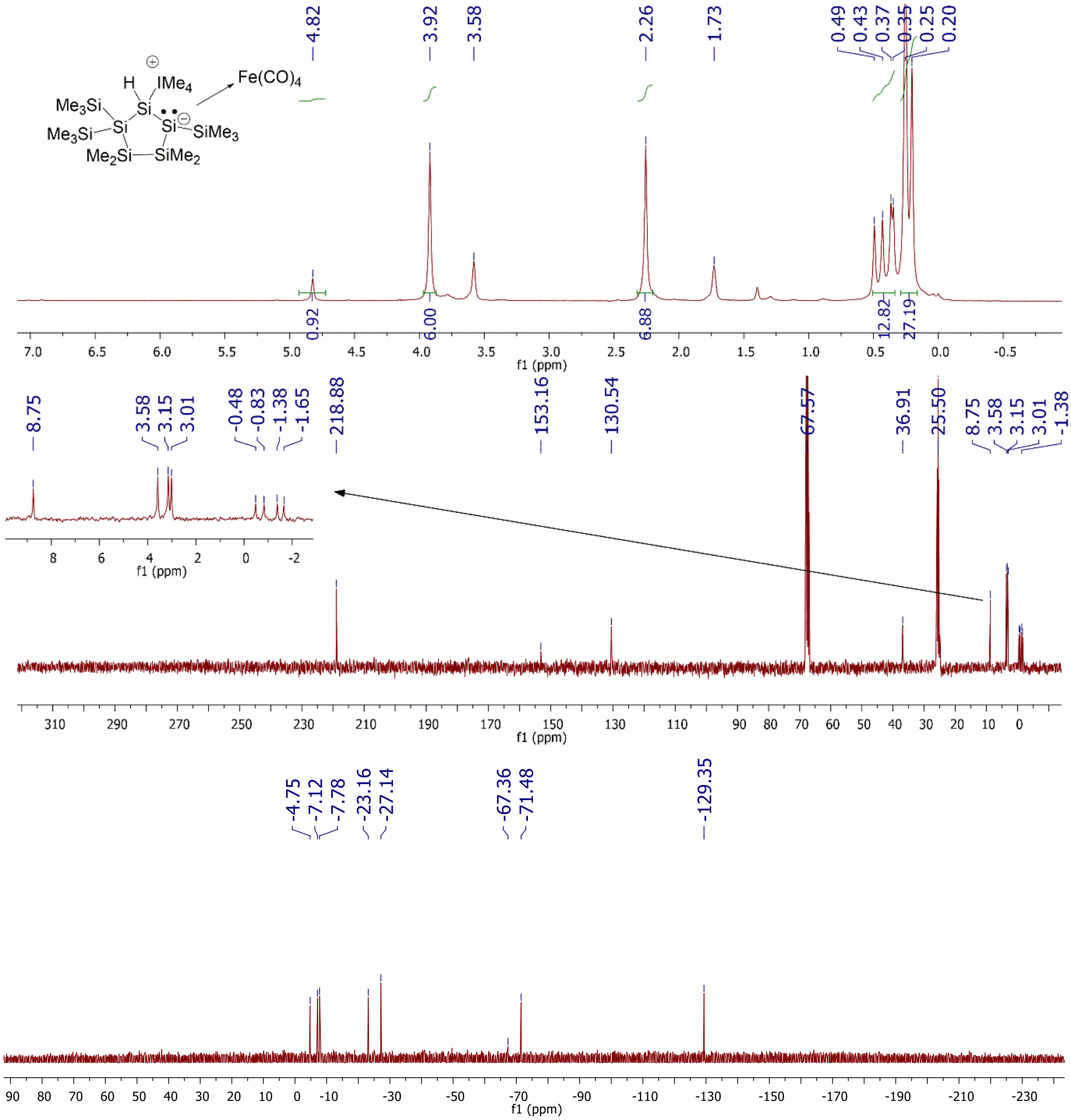

Figure S5: ^{1}H-, ^{29}Si- and ^{13}C-NMR spectra of **5a** (THF d_8 solution, vs ext. TMS, ppm) [* toluene and # *n*-pentane]

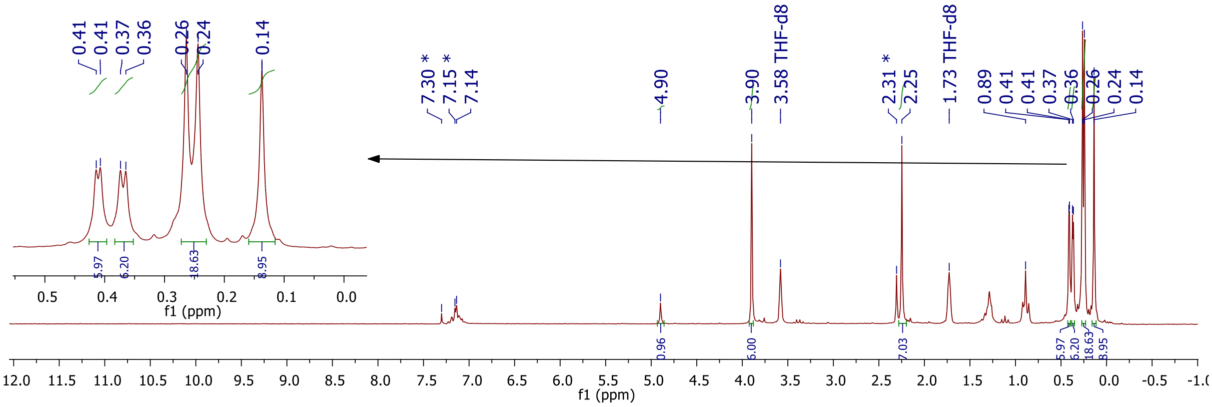

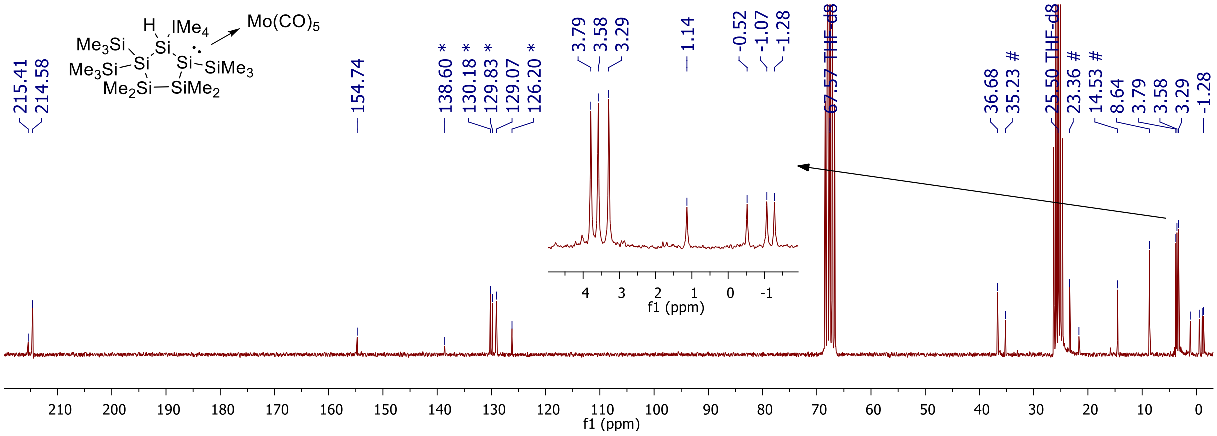

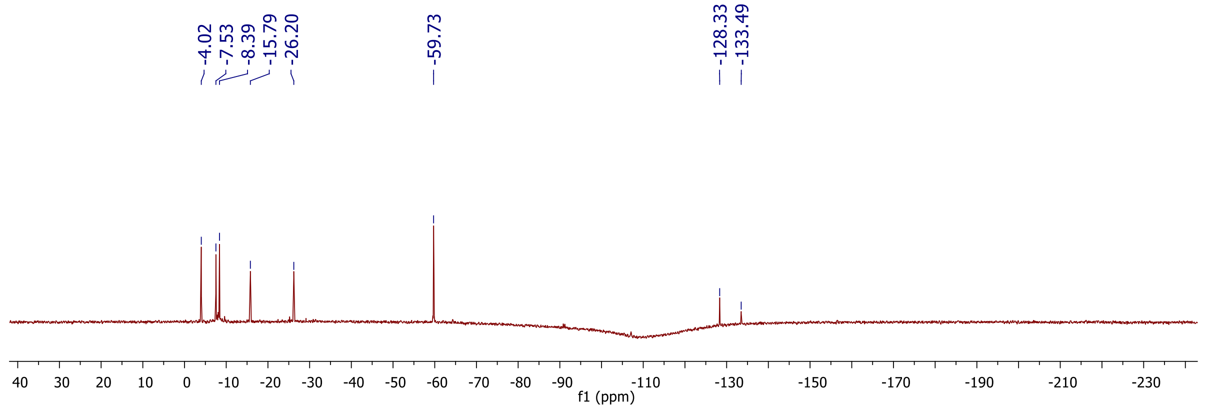

Figure S6: ^{1}H-, ^{29}Si- and ^{13}C-NMR spectra of **5b** (THF d_8 solution, vs ext. TMS, ppm) [* toluene and # *n*-pentane]

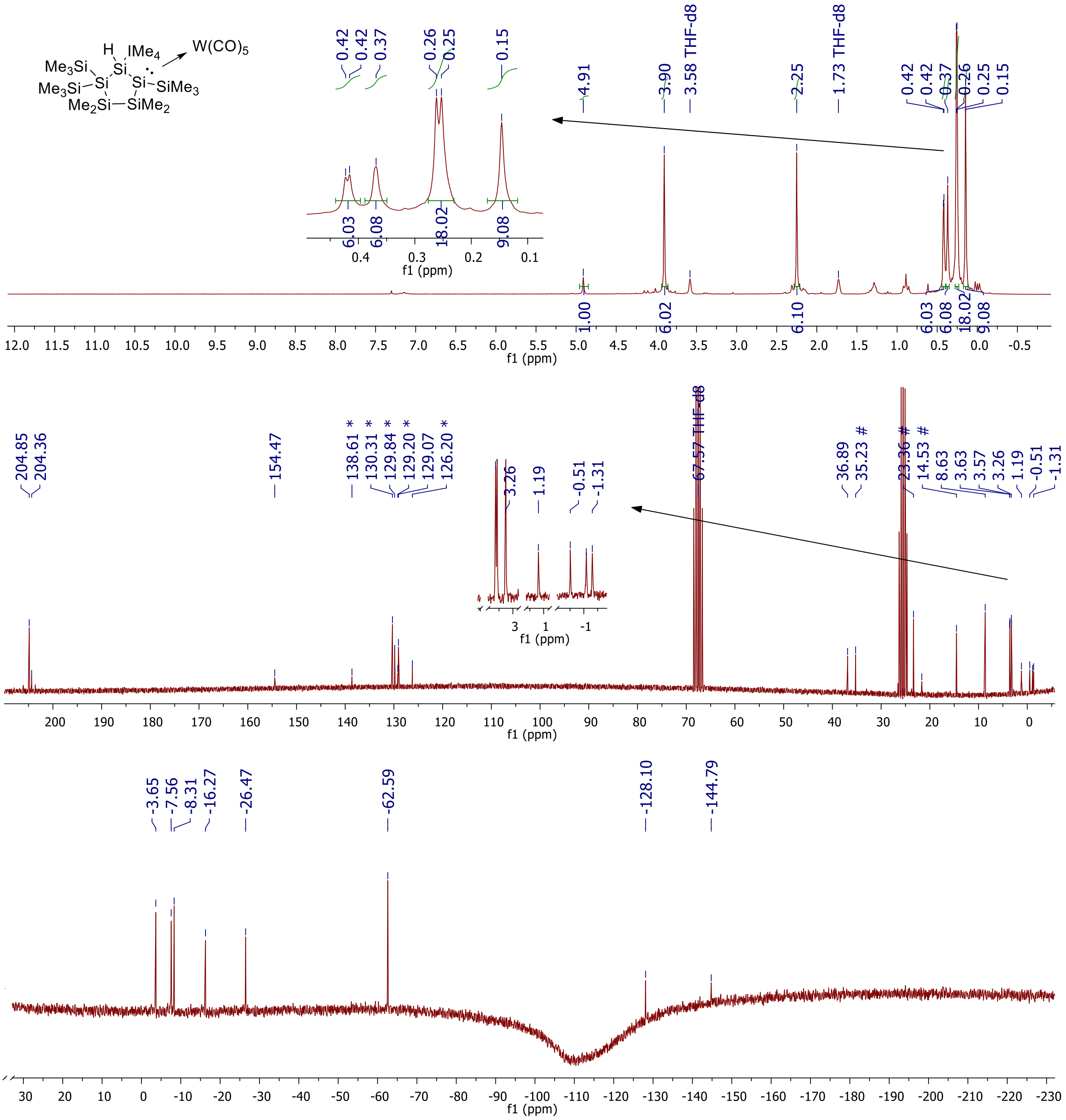

Computational data.

Intermediates of the reaction of 1 with NHCs.

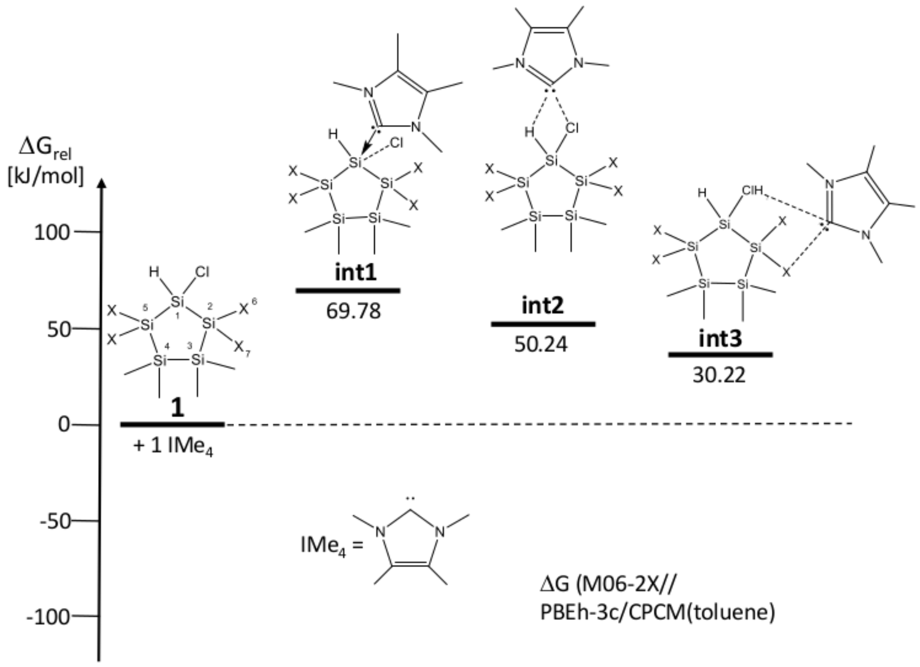

Figure S7. Three stable intermediates were formed when IMe$_4$ approaches from different sides to the educt **1**. **Int1** is discussed in the main paper. The linear reaction path between **Int1** and **Int2** shows a high-energy geometry, but this could not be optimized towards a stable saddle point, as the flexibility of the five-membered ring interferes with the normal mode sampling. In **Int3**, the carbene is lying below the silyl group with a very loose distance (4.195 A) to Si6. The bulky TMS groups prevent a direct attachment of the carbene at Si2, and therefore, a practicable starting point of a silanide formation cannot be formed for our compound with the five-membered ring. Relative Gibbs energies are given in kJ/mol as calculated with M06-2X/def2-TZVP//PBeh-3c in CPCM(toluene).

Reaction Gibbs Energies.

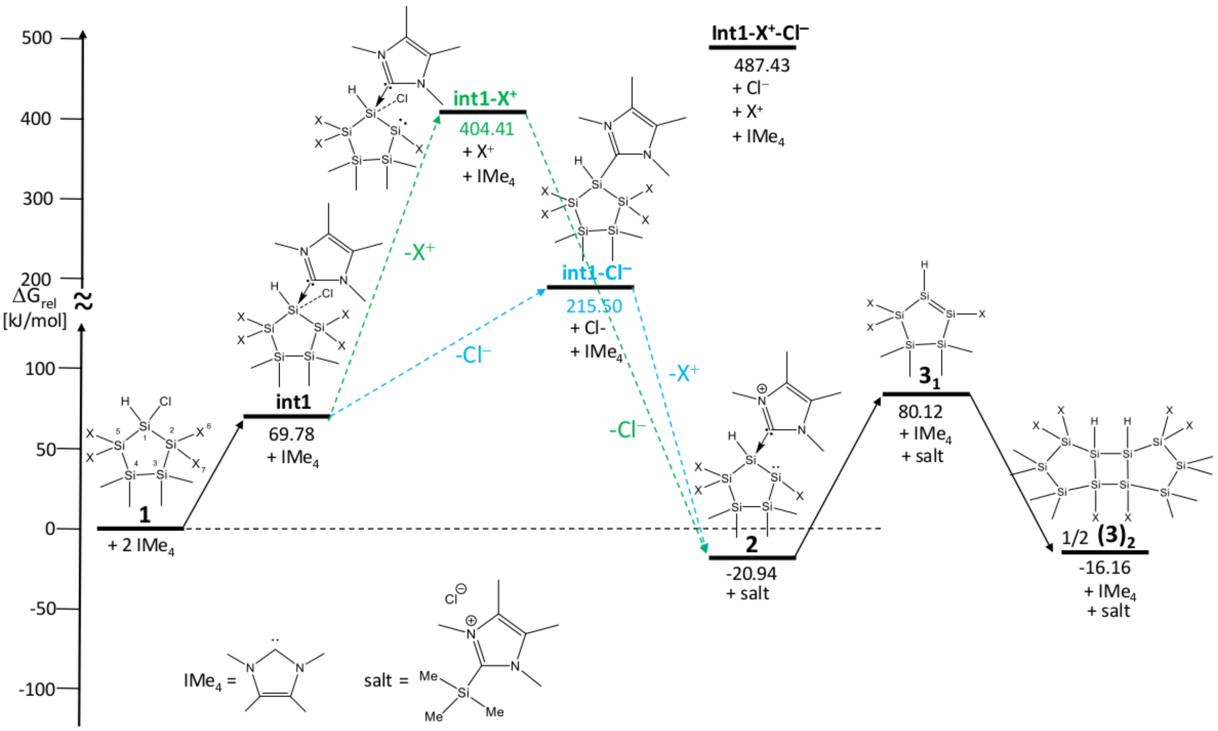

Figure S8. The calculated reaction with Gibbs energies for the stable conformations from educt **1** towards the dimer **3**. In the intermediate **Int1**, the chlorine is loosely bound to Si1, and the NHC IMe_4 has formed a single bond to it. The release of the silyl group from intermediate **Int1** as the first step (green), has a higher energy than the release of chlorine (blue) from Si1. The energies were computed by summing up the individual energies as given in the plot. Overall, product **2** is stabilized (-20.94 kJ/mol) relative to the educt when the salt is formed as a second product. The sum of the Gibbs energies of **2**-**NHC** (the product 2 without stabilization by NHC) with the individual ions plus carbene is much higher indicating that the second carbene molecule plays a crucial role in the mechanism. Calculations were performed with M06-2X/def2-TZVP//PBeh-3c in CPCM(toluene).

Rearrangement of 2 towards silylene.

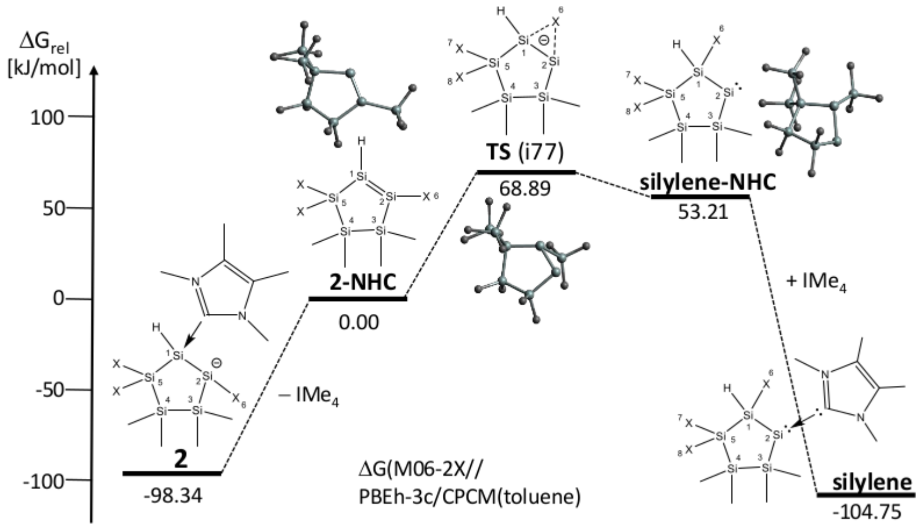

Figure S9. Without the attached NHC IMe_4, **2-NHC** is 98.34 kJ/mol less stable than **2**. Rearrangement of the silyl group to form the respective silylene shows a barrier of 68.89 kJ/mol. The NHC-free **silylene-NHC** is 53.21 kJ/mol less stable than **2**, and it is stabilized by the carbene IMe_4 by 157.96 kJ/mol, which makes the **silylene** by ca. 6 kJ/mol more stable than **2**. Calculations were performed with M06-2X/def2-TZVP//PBeh-3c in CPCM(toluene).

Thermodynamics of the reaction from 1 to 2.

Table S1. Reaction Gibbs Free energy of different abstraction reactions. Relative Gibbs free energies were calculated by different group's abstraction from **1** without the stabilizing NHC IMe_4. Relative Gibbs energies are given in kJ/mol as calculated with M06-2X/def2-TZVP//PBeh-3c in CPCM(toluene). Calculations were performed with M06-2X/def2-TZVP//PBeh-3c in CPCM(toluene).

**1**	→ released +	product2	ΔG_{rel} [a] [/kJ/mol]
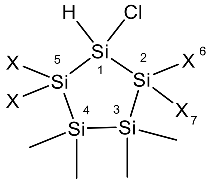	$\rightarrow Cl^- +$	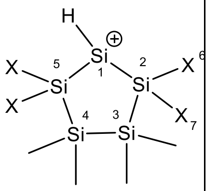	+514.37
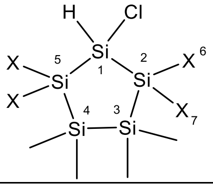	$\rightarrow X^+ +$	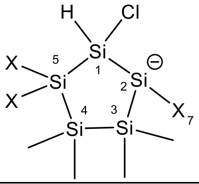	+470.74
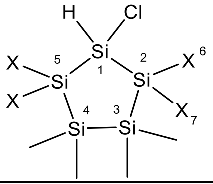	$\rightarrow \mathrm{HCl} +$	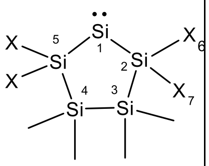	+328.53
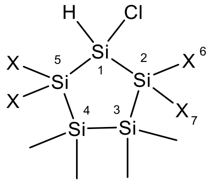	$\rightarrow X^+ + Cl^- +$	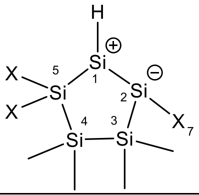	+705.63

[a] $\Delta G_{rel,rxn} = \Sigma_i\,(\Delta G_{i,products}) - \Delta G_{educt\ 1}$

Table S2. Reaction Gibbs Free energy of different abstraction reactions. Relative Gibbs free energies were calculated by the abstraction of different group from **Int1** including bound NHC IMe_4 in all products. Relative Gibbs energies are given in kJ/mol as calculated with M06-2X/def2-TZVP//PBeh-3c in CPCM(toluene). Calculations were performed with M06-2X/def2-TZVP//PBeh-3c in CPCM(toluene).

**int1**	→ released +	product2	ΔG_{rel} ^{a} /kJ/mol
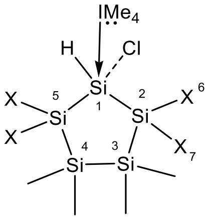	$\rightarrow Cl^- +$	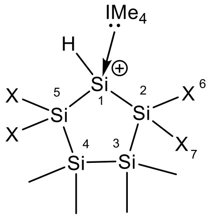	+198.91
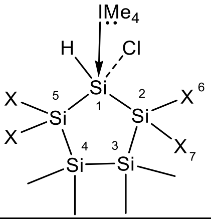	$\rightarrow X^+ +$	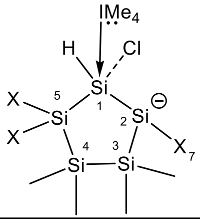	+387.83
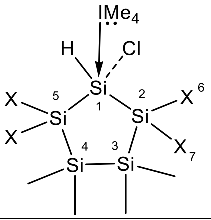	$\rightarrow$ HCl +	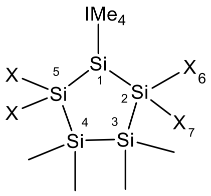	+89.59
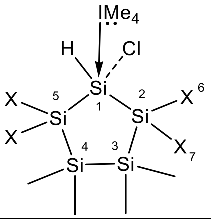	$\rightarrow X^+ + Cl^- +$	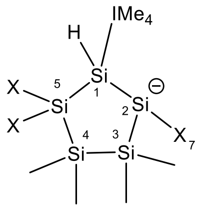	+470.85

^{a} $\Delta G_{rel,rxn} = \Sigma_i\ (\Delta G_{i,products}) - \Delta G_{int1}$

Bonding analysis of 2 compared to Cowley's compound A^{Cp*}.

The bond lengths analysis and the NPA charge distribution of compound **2** is discussed together with a topological analysis of the electron density in the main part of the manuscript and confirms Lewis structure **2c**. Here, the NBO analysis, the Laplacian as well as the bond critical points (bcps) are depicted, which confirm our interpretation.

Compound **2** shows single bonds across the five-membered silicon ring and to the attached TMS groups with small bond length variations between 2.34-2.74 Å. Only the Si1-Si2 bond (2.324 Å) is a bit shorter by 0.04 Å, but this is still in the range of a single bond. The computed NMR shift of Si2 (-229.3 ppm) is comparable with the measured value within 20 ppm (202.66 ppm). The computed negative partial charge at Si2 (-0.525 e) confirms the anionic character of the three-coordinated Si2.

Comparison of **2** with compound $\mathbf{A^{Cp*}}$ shows a very similar bonding behavior. Only atom Si1 is slightly more positive in $\mathbf{A^{Cp*}}$ (0.874 e) than in **2** (0.351 e), but this is justified by the steric situation of the five-membered ring and the different attached moiety (pentamethylcyclopentadienyl in $\mathbf{A^{Cp*}}$, and Si5 with two TMS groups in **2**). The Natural Bonding Orbital (NBO) data also shows a very similar bonding situation in **2** and $\mathbf{A^{Cp*}}$ (see Table S3). The polarization and hybridizations agree well with the exception of the Si1-Si5 bond (which is a Si1-C_{Cp*} bond in $\mathbf{A^{Cp*}}$). The lone pair at Si2 is slightly more occupied in compound **2**, and the Mayer bond valence is a bit reduced (3.02 vs. 3.16) for Si2-Si3 whereas it is slightly higher (3.98 vs. 3.60) for Si1-Si2. Also, the Mayer bond order is a bit smaller for S1-Si2. These data agree well with the reduced NPA charge at the negatively charged Si2 (see the main paper).

The Laplacian of both molecules, **2** and $\mathbf{A^{Cp*}}$, agree well (see Fig. S10) showing a density concentration in the center of the bond with a minimum closer to the more electronegative center. The 5-membered ring is pushing somewhat more electron density into the ring and toward the hydrogen atom in compound **2** (the H-atom attached at Si1 is not shown). The Laplacian value is more positive for the NHC-Si bond (+0.335 $eBohr^{-5}$) compared to the Si1-Si2 bond (0.203 $eBohr^{-5}$) indicating a stronger dative bond for the former. The dative character of both bonds is supported by the position of the bond critical points, which are shifted toward Si1 and Si2, respectively, (see data in Table S4) confirming the higher dative character of both bonds. To conclude, the charge distribution, NBO data and topological analysis confirm the bonding character for the Lewis structure **2c**.

Table S3. Mayer population and NBO analysis of compound 2 (in black) and Cowleys A^{Cp*} (in blue). The respective geometries were optimized with the PBEh-3c method.

NBO analysis	polarization [%] **2** ($\mathbf{A^{Cp*}}$)	Hybridization	occupation	Mayer bond order	Mayer's bond valence
Si1-Si2	59.14% Si1 - 40.86% Si2<br>57.38 % Si1 -42.62% Si2	$sp^{1.99}$ - $sp^{7.10}$<br>$sp^{2.11}$ - $sp^{6.10}$	1.914<br>1.915	0.949<br>0.966	3.98 (Si1)<br>3.60 (Si1)
Si2-Si3	49.30% Si2 – 50.70% Si3<br>50.52 % Si2 -49.48% Si3	$sp^{5.04}$ – $sp^{2.76}$<br>$sp^{5.31}$ – $sp^{2.66}$	1.896<br>1.888	0.961<br>0.975	3.02 (Si2)<br>3.16 (Si2)
Si2-Si6	51.69% Si2 – 48.31% Si6	$sp^{3.18}$ – $sp^{23.36}$	1.925	0.947	3.98 (Si6)

	52.03 % Si2 -47.97% Si6	$sp^{4.65} – sp^{2.780}$	1.903	0.966	3.60 (Si6)
Si1-Si5/C(Cp*)	48.46% Si1 – 51.54% Si5<br>27.66 % Si1 -72.34% Si2	$sp^{1.99}$ - $sp^{7.10}$<br>$sp^{2.70} – sp^{4.60}$	1.914<br>1.847	0.949<br>0.768	
Si1-H	43.82% Si1 – 56.18% H<br>41.96% Si1 -58.04% H	$sp^{1.99}$ - $sp^{7.10}$<br>$sp^{2\cdot 11}$ - $sp^{6.10}$	1.914<br>1.962	0.949<br>0.966	
Si1-C(NHC)	26.69% Si1 – 73.31% C<br>25.71% Si1 -74.29% C	$sp^{4.19} – sp^{1.26}$<br>$sp^{4.43} – sp^{1.29}$	1.960<br>1.957	0.764<br>0.745	3.40 (C)<br>3.37 (C)
LP(Si2)		$sp^{0.85}$<br>$sp^{0.90}$	1.514<br>1.798		

Table S4. Topological data of the electron density of **2**: Laplacian $\nabla^2\rho(r)$ (in eBohr^{-5}), bonding distance and position of the bond critical points (bcp) (in Å) and total energy density H(r) (in E_HBohr^{-3}). The program Multiwfn was applied for these data

NBO analysis	$\nabla^2\rho$	d_{A-B}	d_{A_bcp}	d_{bcp_B}	H_{bcp}
Si1-Si2	+0.203	2.329	0.832	1.497	-0.044
Si2-Si3	-0.560	2.352	0.855	1.497	-0.051
Si2-Si6	-0.546	2.349	0.847	1.502	-0.053
Si1-Si5	-0.968	2.364	1.485	0.879	-0.053
Si1-H	+0.273	1.497	0.730	0.767	+0.058
Si1-C(NHC)	+0.325	1.939	0.741	1.198	-0.039

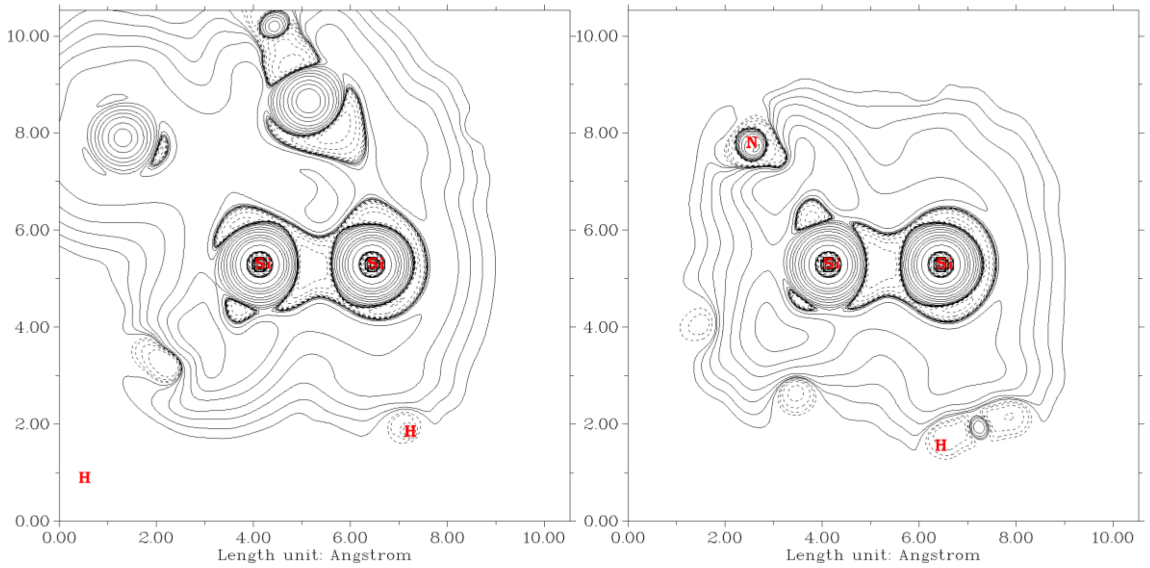

Figure S10. 2D plot of the Laplacian $\nabla^2\rho(r)$ for **2** (left) and $\mathbf{A}^{\mathbf{Cp*}}$ (right), both normal to the C_{NHC}-Si1-Si2 plane. Contour plots are drawn with the program MultiWfn.

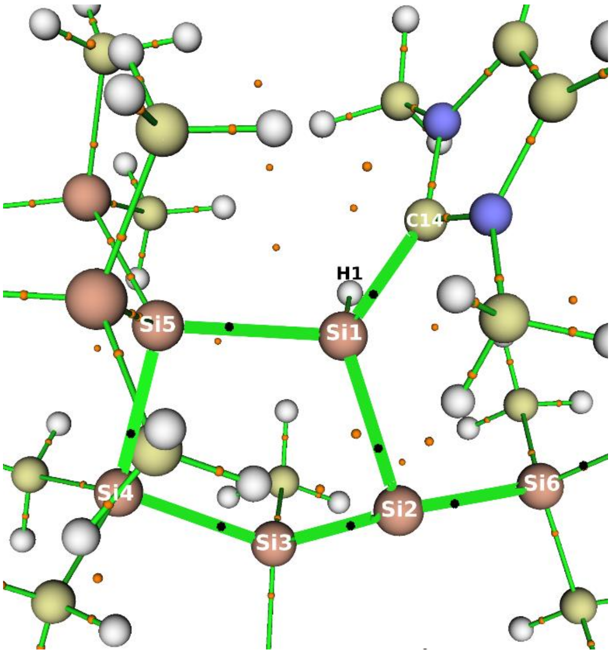

Figure S11. Bond critical points (bcps) of **2** in orange; important bcps are intensified in black for better visibility. The bonds in the five-membered ring and the Si-C bond towards the NHC are intensified for a better visibility.

UV/Vis data of 2.

The molecular orbitals of the first two excitations are depicted in the main part of the manuscript and the vertical excitation data are collected in Table S5.
The first two excitations occur from the Si1 p_z orbital to the carbene (S1) and to the silicon five-membered ring (S2). The calculated spectrum agrees perfectly with the experimental UV/Vis spectrum of **2**. Figure S12 depicts the simulated absorption spectrum in the spectral region from 250-600 nm.

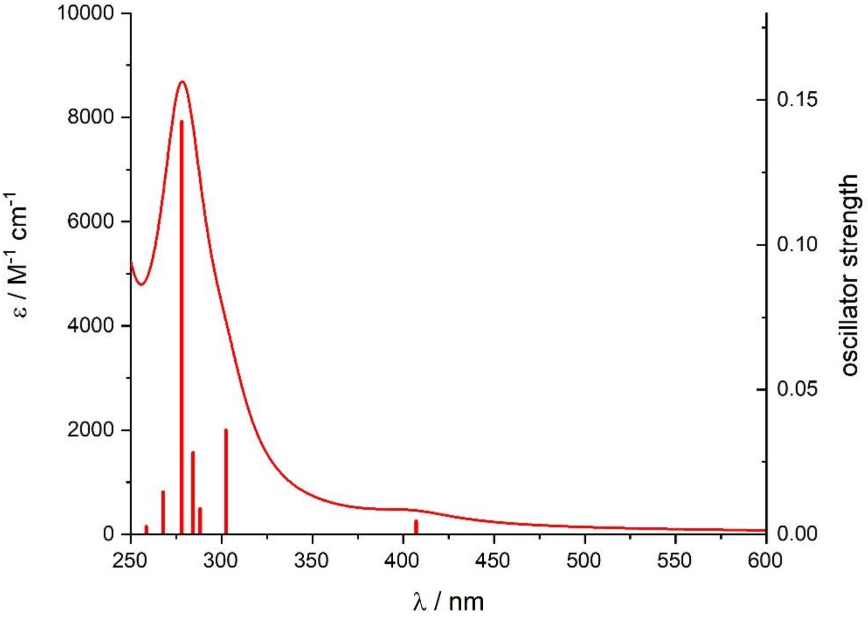

Figure S12. Calculated vertical excitations of **2,** and simulated spectrum with a FWHH broadening of 3600 cm^{-1}.

Table S5. First three calculated vertical excitations of **2**. The orbitals refer to the MO picture in the main part of the manuscript.

	λ / nm	E / eV	f	orbital contributions (LCAO coefficients c^2)
S_1	407.1	3.046	0.0046	H $\rightarrow$ L (0.99)
S_2	302.3	4.102	0.0360	H $\rightarrow$ L+1 (0.87)
S_3		4.301	0.0088	H $\rightarrow$ L+3 (0.49),<br>H $\rightarrow$ L+2 (0.31)

NMR chemical shifts of 2.

The following chemical shifts were computed for **2** in benzene and agree well within 20 ppm with the experimental data.

Table S6. Comparison of calculated and experimental NMR shifts (in ppm).

**2**	DFT chem. shift	exp. chem. shift
Si1	-61.3	-71.5
Si2	-229.3	202.66
Si3	-23.7	-13.59
Si4	-33.7	-21.98
Si5	-143.0	130.94
Si6	-7.6	-3.93
Si5’	-14.0	-8.88
Si5’’	-13.6	-9.72
H1	5.1	5.2
$C_{carbene}$	168.4	157.87

Crystallographic Tables

Identification code	**2**	**3**	**4**	**5a**	**5b**
CCDC	2115666	2115667	2115668	2115669	2115670
Empirical formula	$C_{10}H_{26}NSi_4$	$C_{26}H_{80}Si_{16}$	$C_{27.5}H_{56}FeN_2O_4Si_8$	$C_{27}H_{56}MoN_2O_{5.5}Si_8$	$C_{25}H_{52}N_2O_5Si_8W$
Formula weight	272.68	842.34	759.31	817.39	869.25
Temperature/K	99.99	100.03	100.03	100.03	99.99
Crystal system	triclinic	monoclinic	triclinic	monoclinic	monoclinic
Space group	P-1	C2/c	P-1	C2/c	$P2_1/n$
a/Å	11.2266(5)	26.2840(11)	11.6554(4)	21.1291(9)	11.3203(5)
b/Å	11.6051(5)	12.1048(5)	11.8956(5)	11.9107(4)	20.6352(9)
c/Å	14.4389(7)	17.9037(7)	17.7486(7)	34.2777(11)	17.8171(7)
α/°	69.394(2)	90	77.709(2)	90	90
β/°	76.124(2)	112.913(2)	84.466(2)	97.675(2)	101.496(2)
γ/°	72.908(2)	90	61.770(2)	90	90
Volume/Å^3	1663.39(13)	5246.8(4)	2118.39(15)	8549.1(5)	4078.5(3)
Z	4	4	2	8	4
ρ_{calc}g/cm^3	1.089	1.066	1.190	1.270	1.416
μ/mm^{-1}	0.334	0.405	0.613	0.566	3.099
F(000)	596.0	1840.0	810.0	3440.0	1768.0
Crystal size/mm^3	0.21×0.18×0.13	0.27×0.16×0.07	0.17×0.14×0.1	0.24×0.13×0.1	0.15×0.12×0.08
Radiation	MoK$_\alpha$ (λ = 0.71073)	MoK$_\alpha$ (λ = 0.71073)	MoK$_\alpha$ (λ = 0.71073)	MoK$_\alpha$ (λ = 0.71073)	MoK$_\alpha$ (λ = 0.71073)
2Θ range for data collection/°	3.05 to 57.814	3.364 to 60.028	4.098 to 56.204	3.89 to 55.788	3.938 to 60.238
Index ranges	-14 ≤ h ≤ 15, -15 ≤ k ≤ 15, -19 ≤ l ≤ 19	-36 ≤ h ≤ 36, -17 ≤ k ≤ 17, -24 ≤ l ≤ 25	-15 ≤ h ≤ 15, -15 ≤ k ≤ 15, -22 ≤ l ≤ 23	-27 ≤ h ≤ 27, -15 ≤ k ≤ 15, -45 ≤ l ≤ 45	-15 ≤ h ≤ 15, -29 ≤ k ≤ 29, -25 ≤ l ≤ 25
Reflections collected	16225	104488	64462	205300	208352
Independent reflections	8709 R_{int} = 0.0344, R_{sigma} = 0.0515	7635 R_{int} = 0.0678, R_{sigma} = 0.0433	10255 R_{int} = 0.0650, R_{sigma} = 0.0614	10142 R_{int} = 0.1130, R_{sigma} = 0.0446	11982 R_{int} = 0.0603, R_{sigma} = 0.0232
Data/restraints/parameters	8709/126/373	7635/0/207	10255/46/437	10142/35/435	11982/0/390
Goodness-of-fit on F^2	1.141	1.070	1.034	1.103	1.031
Final R indexes [I>=2σ (I)]	R_1 = 0.0505, wR_2 = 0.1264	R_1 = 0.0420, wR_2 = 0.0867	R_1 = 0.0389, wR_2 = 0.0798	R_1 = 0.0491, wR_2 = 0.1313	R_1 = 0.0179, wR_2 = 0.0372
Final R indexes [all data]	R_1 = 0.0632, wR_2 = 0.1306	R_1 = 0.0751, wR_2 = 0.1021	R_1 = 0.0757, wR_2 = 0.0922	R_1 = 0.0548, wR_2 = 0.1350	R_1 = 0.0237, wR_2 = 0.0393
Largest diff. peak/hole / e Å^{-3}	0.49/-0.60	1.10/-0.46	0.50/-0.39	1.54/-1.18	0.74/-0.79

References

[1] A. B. Pangborn, M. A. Giardello, R. H. Grubbs, R. K. Rosen, F. J. Timmers, *Organometallics* 1996, 15,1518

[2] a) F. Höfler, R. Jannach, *Z. anorg. Allg. Chem*. 1975, 413, 285-292 b) Zhou, Xiaobing, (Dow Corning Corporation), WO 2012125432

[3] Mankad, N. P.; Laitar, D. S; Sadighi, J. P. Synthesis, Structure, and Alkyne Reactivity of a Dimeric (Carbene)copper(I) Hydride, *Organometallics* 2004, 23, 3369-3371

[4] Frisch, M. J.; Trucks, G. W.; Schlegel, H. B.; Scuseria, G. E.; Robb, M. A.; Cheeseman, J. R.; Scalmani, G.; Barone, V.; Mennucci, B.; Petersson, G. A.; Nakatsuji, H.; Caricato, M.; Li, X.; Hratchian, H. P.; Izmaylov, A. F.; Bloino, J.; Zheng, G.; Sonnenberg, J. L.; Hada, M.; Ehara, M.; Toyota, K.; Fukuda, R.; Hasegawa, J.; Ishida, M.; Nakajima, T.; Honda, Y.; Kitao, O.; Nakai, H.; Vreven, T.; Montgomery, Jr., J. A.; Peralta, J. E.; Ogliaro, F.; Bearpark, M.; Heyd, J. J.; Brothers, E.; Kudin, K. N.; Staroverov, N.; Keith, T.V.; Kobayashi, R.; Normand, J.; Raghavachari, K.; Rendell, A.; Burant, J. C.; Iyengar, S. S.; Tomasi, J.; Cossi, M.; Rega, N.; Millam, J. M.; Klene, M.; Knox, J. E.; Cross, J. B.; Bakken, V.; Adamo, C.; Jaramillo, J.; Gomperts, R.; Stratmann, R. E.; Yazyev, O.; Austin, A. J.; Cammi, R.; Pomelli, C.; Ochterski, J. W.; Martin, R. L.; Morokuma, K.; Zakrzewski, V. G.; Voth, G. A.; Salvador, P.; Dannenberg,

J. J.; Dapprich, S.; Daniels, A. D.; Farkas, O.; Foresman, J. B.; Ortiz, J. V.; Cioslowski, J.; Fox, D. J.; Gaussian 09, Revision D.01, Gaussian, Inc., Wallingford CT, **2013**.
[5] Becke, A. *J. Chem. Phys.* **1993**, 98, 1372.
[6] Lee, C.; Yang, W.; Parr, R. Phys. Rev. B: *Condens. Matter Mater. Phys.* **1988**, 37, 785−789.
[7] a) Hariharan, P. C.; Pople, *J. A. Theor. Chim. Acta* **1973**, 28, 213−222; b) Hehre, W. J.; Ditchfield, R.; Pople, *J. A. J. Chem. Phys.* **1972**, 56, 2257−2261; c) Ditchfield, R.; Hehre, W. J.; Pople, *J. A. J. Chem. Phys.* **1971**, 54, 724−728.
[8] Tomasi, J.; Mennucci, B.; Cammi, R. *Chem. Rev.* **2005**, *105*, 2999-3093.
[9] Allouche, *A. R. J. Comput. Chem.* **2011**, 32, 174-182.
[10] Bruker: APEX2 and SAINT. Bruker AXS Inc.: Madison, Wisconsin, USA, **2012**.
[11] Blessing, R.; *Acta Crystallogr., Sect. A* **1995**, *51*, 33-38.
[12] Sheldrick, G.; *Acta Crystallogr., Sect. A* **1990**, *46*, 467-473.
[13] Sheldrick, G.; *Acta Crystallogr., Sect. A* **2008**, *64*, 112-122.
[14] Sheldrick, G.; *Acta Crystallogr., Sect. A: Found. Adv.* **2015**, *71*, 3-8.
[15] Spek, A. L.; *J. Appl. Crystallogr.* **2003**, *36*, 7-13.
[16] Spek, A. L.; *Acta Crystallogr., Sect. D* **2009**, *65*, 148-155.
[17] Dolomanov, O. V.; Bourhis, L. J.; Gildea, R. J.; Howard, J. A. K.; Puschmann,H.; *J. Appl. Crystallogr.* **2009**, *42*, 339–341.
